# Massive introgression drives species radiation at the range limit of *Anopheles gambiae*

**DOI:** 10.1038/srep46451

**Published:** 2017-04-18

**Authors:** José L. Vicente, Christopher S. Clarkson, Beniamino Caputo, Bruno Gomes, Marco Pombi, Carla A. Sousa, Tiago Antao, João Dinis, Giordano Bottà, Emiliano Mancini, Vincenzo Petrarca, Daniel Mead, Eleanor Drury, James Stalker, Alistair Miles, Dominic P. Kwiatkowski, Martin J. Donnelly, Amabélia Rodrigues, Alessandra della Torre, David Weetman, João Pinto

**Affiliations:** 1 Global Health and Tropical Medicine, GHTM, Instituto de Higiene e Medicina Tropical, IHMT, Universidade Nova de Lisboa, UNL, Lisbon, Portugal; 2 Department of Vector Biology, Liverpool School of Tropical Medicine, Liverpool, United Kingdom; 3 Istituto Pasteur Italia-Fondazione Cenci-Bolognetti, Dipartimento di Sanità Pubblica e Malattie Infettive, Università di Roma “Sapienza”, Rome, Italy; 4 Instituto Nacional de Saúde Pública, Ministério da Saúde Pública, Bissau, Guiné-Bissau; 5 Wellcome Trust Centre for Human Genetics, University of Oxford, Oxford, United Kingdom; 6 Istituto Pasteur Italia-Fondazione Cenci-Bolognetti, Dipartimento di Biologia e Biotecnologie “Charles Darwin”, Università di Roma “Sapienza”, Rome, Italy; 7 Wellcome Trust Sanger Institute, Hinxton, UK; 8 Medical Research Council Centre for Genomics and Global Health, University of Oxford, Oxford, UK

## Abstract

Impacts of introgressive hybridisation may range from genomic erosion and species collapse to rapid adaptation and speciation but opportunities to study these dynamics are rare. We investigated the extent, causes and consequences of a hybrid zone between *Anopheles coluzzii* and *Anopheles gambiae* in Guinea-Bissau, where high hybridisation rates appear to be stable at least since the 1990s. *Anopheles gambiae* was genetically partitioned into inland and coastal subpopulations, separated by a central region dominated by *A. coluzzii*. Surprisingly, whole genome sequencing revealed that the coastal region harbours a hybrid form characterised by an *A. gambiae*-like sex chromosome and massive introgression of *A. coluzzii* autosomal alleles. Local selection on chromosomal inversions may play a role in this process, suggesting potential for spatiotemporal stability of the coastal hybrid form and providing resilience against introgression of medically-important loci and traits, found to be more prevalent in inland *A. gambiae*.

Interspecific hybridisation is an important, yet probably underestimated, force in the evolution of animal biodiversity with possible impacts ranging from erosion of species divergence and ultimate species collapse^1^ to rapid adaptation or even speciation^2^,^3^. The latter ‘hybrid speciation’ is predicted to arise via accumulation of genetic variation through introgression, resulting in admixed populations that are ecologically divergent from the parental lineages^3^. More generally, whilst rare hybridization can undoubtedly lead to adaptive introgression^4^,^5^, the adaptive potential of very frequent interspecific hybridization is far less clear. Systems in which uncharacteristically extreme hybridisation rates are found offer the opportunity to study such evolutionary processes. The *Anopheles coluzzii-Anopheles gambiae* malaria vector species-pair in western Africa provides an ideal opportunity to study speciation under extreme hybridization^6^,^7^.

Members of the *Anopheles gambiae* complex include the most important malaria vectors in Sub-Saharan Africa. Early evidence of genetic discontinuities in West Africa within the nominal species of the complex, *A. gambiae*, came from cytogenetic studies, which led to the description of five chromosomal forms, the FOREST and SAVANNA forms widespread in west, central and east Africa, the MOPTI form in northern west African savannah areas, and the BAMAKO and BISSAU forms, which are found only in localized areas of Mali and the far-west coast, respectively. Each is characterized by different arrangements of paracentric inversions on chromosome-2 and differing larval ecologies^8^,^9^, and studies have associated specific inversion polymorphisms with multiple environmental adaptions^8^,^9^,^10^. However, following the discovery of fixed differences in ribosomal DNA on the pericentromeric region of chromosome-X, which overlapped only partially with the chromosomal forms, focus shifted from chromosomal to ‘molecular forms’, termed M and S^11^. These forms have been recently named *Anopheles coluzzii* and *A. gambiae*^12^, respectively and the species-pair has become a model system for speciation with gene flow^13^ due to their closely related genomes^14^ characterized by three large highly divergent ‘genomic islands’ near the centromeres of each chromosome^15^,^16^. Subsequent analyses have shown that divergence is more widespread around the genome than first thought^5^,^17^ and the involvement of two of the three islands of divergence in early-stage speciation became controversial^5^,^18^,^19^,^20^. However, a recent laboratory study has demonstrated that the chromosome-X island of divergence is associated with assortative mating between *A. coluzzii* and *A. gambiae*^21^ confirming this genomic region as a primary candidate location for genes involved in reproductive isolation. These species have also shown differences in at least two genes which contribute to medically important phenotypes. First, mutations associated with knockdown resistance (*kdr*) to DDT and pyrethroid insecticides have been initially found at much higher frequency in *A. gambiae* than in *A. coluzzii* even when the species are sympatric^22^. There is evidence that recent adaptive introgression has subsequently levelled *kdr* frequencies between species^5^,^20^,^23^, although this imbalance appears to persist in the far western region^24^. Second, the allelic variant *r1* of the immune-related *tep1* gene, which has been linked to resistance to infection by *Plasmodium falciparum* malaria parasites, was found to be exclusive to *A. coluzzii*^25^.

Reproductive isolation between *A. coluzzii* and *A. gambiae* occurs at the adult stage by incompletely-understood pre-mating mechanisms^26^, while no intrinsic postzygotic isolating mechanisms are observed between the two species^27^. At the larval stage, ecological niche partitioning and selection against hybrids are likely to play important roles in species segregation^28^. *Anopheles gambiae* typically breeds in rain-dependent ephemeral pools, while *A. coluzzii* is more successful in breeding sites created by irrigation (*e.g.* rice-fields) or other human activities (*e.g.* water reservoirs), or in urban polluted environments^29^. There is also evidence that *A. coluzzii* larvae display greater salinity tolerance, which may permit better exploitation of marginal habitats^30^. Despite broad sympatry between *A. coluzzii* and *A. gambiae* across West and Central Africa, hybrid rates, as detected by X-linked species-species diagnostic markers^11^,^31^, are usually below 1%^32^. However, assortative mating may periodically be disrupted and temporally-unstable bursts of elevated hybridisation have been reported^19^. The ‘typical’ situation of low hybridisation breaks down most clearly in a hybrid zone between *A. coluzzii* and *A. gambiae* on the western edge of the species’ distribution^6^,^33^. Although, as yet undetected, stable high hybridisation may occur elsewhere, the far-west region has become an important case study area, with hybridisation rates >20% being recorded persistently for almost 20 years in Guinea Bissau^6^,^7^,^34^. Hybridization appears to involve asymmetric introgression from *A. coluzzii* to *A. gambiae*^7^ and to have eroded the major genomic islands of divergence on chromosomes-2 and -3 but to a far lesser extent on chromosome-X^19^,^35^,^36^,^37^.

The temporal stability and spatial extent of the hybrid zone are of both academic interest and medical concern. Whilst continued introgression might ultimately lead to erosion of divergence or even species collapse^1^ in the hybrid zone, available evidence from the analysis of temporal samples in the area is equivocal. Gordicho *et al*.^34^ pointed to an apparent decline of *A. coluzzii* in coastal Guinea Bissau, from a frequency of 23–44% between 1993 and 1996 to 8.0% and 4.5% in 2007 and 2010, respectively. However, a frequency close to 25% was recorded for *A. coluzzii* in a sample collected in the same locality in 2009^7^, indicating that stochastic temporal instability cannot be ruled out. Moreover, the available temporal data is based on a relatively limited number of collections and without accounting for eventual seasonal variations.

Another possibility is that introgression may be creating a pool of aberrantly high diversity facilitating adaptation to new or marginal niches^2^,^3^. Intriguingly, the hybrid zone overlaps with one of the best examples of intraspecific structuring based on inversion polymorphisms. The BISSAU chromosomal form (characterized by a low frequency of 2Rb and 2La arrangements, and a high frequency of 2Rd) and the SAVANNA chromosomal form (characterized by high frequency of 2Rb and 2La, sometimes with increasing complexity of karyotypes due to the presence of other inversions such as 2Rj and 2Rd) were shown to intergrade from coastal areas to inland, suggesting ecologically-driven genetic divergence due to adaptation to coastal habitats (*e.g.* brackish water habitat and/or competition with the euryhaline sympatric *Anopheles melas* sibling species)^8^,^38^,^39^,^40^.

Other major uncertainties remain about the extreme hybrid zone and its significance. Though documented in neighbouring countries^33^,^41^, the spatial extent of the hybrid zone is still unclear, as are the links to ecology and the impact on traits of medical importance. Although a significantly higher *Plasmodium* infection rate was detected in *A. gambiae* when compared with *A. coluzzii* and hybrids in coastal Guinea Bissau^42^, the potential impact of the hybrid zone on *Plasmodium* transmission and other traits of medical importance, such as insecticide resistance and host preferences, has received limited attention.

In this study, we examined the following hypotheses: (1) The hybrid zone is confined to the coastal region and differentiated from inland regions at chromosomal inversion polymorphisms, which may link to local adaptation; (2) Asymmetric introgression from *A. coluzzii* to *A. gambiae* is promoting intraspecific divergence between coastal and inland populations of *A. gambiae*, potentially leading to radiation of a distinct hybrid form; (3) The establishment of a hybrid form builds the potential for aggregation of medically important mutations and phenotypes.

To investigate these hypotheses, we conducted: (i) a countrywide southwest-northeast transect study using species-specific molecular markers and microsatellites spanning coastal to far-inland Guinea-Bissau, in order to quantify the extent of the hybrid zone; (ii) a focal investigation of chromosomal inversion polymorphisms, in order to determine karyotype differences between coast and inland; (iii) a focal analysis by whole genome sequencing and ancestry informative markers of individuals classified as *A. gambiae*, to determine intraspecific genome-wide divergence between coastal and inland populations of this species and including a comparison with a geographically distant *A. gambiae* sample from a low hybridisation region; (iv) an investigation of markers and traits important for malaria transmission and control in *A. gambiae* from coast and inland.

## Results

### Species distribution and admixture along the transect follow ecological zonation

A total of 687 specimens collected in eight localities of Guinea Bissau were analysed using 19 microsatellite loci (Supplementary Table 1). Polymorphism at chromosome-3 loci was higher than chromosome-X loci, whether measured as allelic richness (*Ar*) or expected heterozygosity (*H*_*e*_) (Mann-Whitney Tests: *Ar*: z = 4.24, *P* < 0.001; *H*_*e*_: z = 4.35, *P* < 0.001) (Supplementary Table 2). None of the loci showed a significant heterozygote deficit across collection sites, which could indicate null alleles, and there was no consistent signal across loci, which could indicate within-sample substructure.

**Figure 1:**
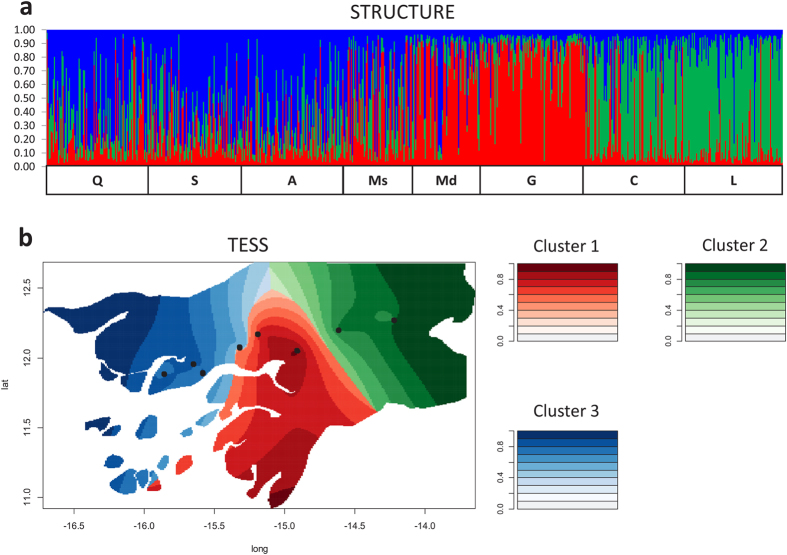
Microsatellite-based Bayesian clustering (STRUCTURE) and spatially explicit analysis (TESS). (**a**) Graph of individual assignment probabilities of belonging to each of the three clusters detected by STRUCTURE. Red: cluster 1 (*A. coluzzii*), green: cluster 2 (*A. gambiae-*inland), blue: cluster 3 (*A. gambiae*-coast). The lower squares delimit mosquito samples according to collection site, from coast to inland (Q: Quinhamel, S: Safim, A: Antula, Ms: Mansoa, Md: Mandingará, G: Ga-Mbana, C: Comuda, L: Leibala). (**b**) Map of Guinea Bissau showing assignment probability densities for the optimal number of clusters obtained in TESS. The map was created by the R-script of software POPS version 1.2^62^ (http://membres-timc.imag.fr/Olivier.Francois/pops.html). Black dots represent the localities sampled.

**Figure 2:**
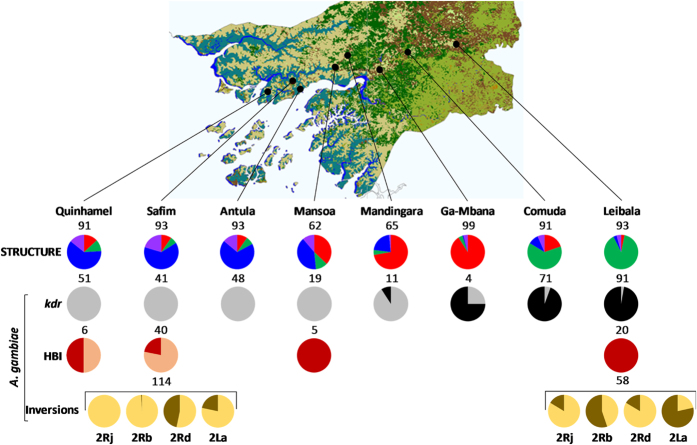
Map of localities surveyed showing microsatellite-based genetic cluster composition (STRUCTURE), and *kdr* frequency, human blood index and chromosome inversion frequency for *Anopheles gambiae*. Map: GlobCover 2009 land cover map of Guinea Bissau. Copyright notice: © ESA 2010 and UCLouvain. Available at: http://www.fao.org/geonetwork/srv/en/metadata.show?id=37189&currTab=simple (Date of access: 27/11/2015). STRUCTURE: proportion of individuals assigned to each genetic cluster (assignment threshold *T*_*q*_ = 0.5). Red: cluster 1 (*A. coluzzii*), green: cluster 2 (*A. gambiae-*inland), blue: cluster 3 (*A. gambiae*-coast), purple: admixed. *kdr*: allele frequency of the knockdown resistance-associated 1014F allele (in black); HBI: human blood index (in red); inversions: frequency of the inverted arrangement in dark yellow at each of four inversions scored. Sample sizes are above each pie chart. Data on *kdr*, HBI and inversions are shown for *A. gambiae* only, identified by IGS/SINE at each site.

Both spatial (TESS) and non-spatial (STRUCTURE) clustering analyses partitioned the overall sample into three distinct genetic clusters (Fig. 1, Supplementary Fig. 1). These clusters differed in their species composition (Table 1) and geographic distribution (Fig. 2). Cluster 1 (hereafter referred to as cluster “*A. coluzzii*”) was dominated by *A. coluzzii* and was located in the central region of the country. In contrast, cluster 2 (referred to as “*A. gambiae*-inland”) was dominated by *A. gambiae* and prevailed in the two eastern inland localities (Comuda and Leibala). Cluster 3 (referred to as “*A. gambiae*-coast”) was comprised almost entirely of *A. gambiae* and IGS/SINE-admixed individuals, and was found mostly in the three western coastal localities (Quinhamel, Antula and Safim). The degree of cross-cluster genetic admixture also varied geographically (Fig. 2). The proportion of admixed individuals (*i.e.* those with *qi* < 0.50 for all clusters determined by STRUCTURE) was higher in coastal localities (14.3–20.4%) than in the central part of the country (1.5–11.3%) and in the eastern inland localities (4.3–6.6%).

### Chromosomal form composition contrasts between coastal and inland samples

A total of 260 half-gravid females collected in coastal Safim (*N* = 202) and inland Leibala (*N* = 58) were karyotyped successfully (Fig. 2, Table 2). *Anopheles gambiae* from Safim were characterized by five karyotypes on chromosome 2R, based on the 2Rb and 2Rd polymorphisms, while the sample from Leibala comprised of 10 karyotypes based on 2Rb, 2Rd inversions and exclusive presence of 2Rj inversion. The high frequencies of 2Rb and 2La inverted arrangements and the high number of karyotypes observed in *A. gambiae* from Leibala (Fig. 2, Table 2) suggest predominance of the SAVANNA chromosomal form, common across West Africa^8^,^9^. Conversely, the higher frequency of the 2 Rd inverted arrangement (χ^2^ = 42.06, d.f. = 2, *P* < 0.001; Fig. 1) coupled with the lower frequency of 2La (χ^2^ = 92.12, d.f. = 2, *P* < 0.001) and the virtual lack of 2Rb (χ^2^ = 126.28, d.f. = 2, *P* < 0.001) inverted arrangements observed in Safim is consistent with predominance of the BISSAU chromosomal form^8^,^9^, occurring in both *A. coluzzii* and *A. gambiae*, which exhibited similar 2Rd and 2La frequencies (Fisher’s exact-test, *P* = 0.23 and *P* = 0.71, respectively).

### Whole genome sequencing reveals extensive autosomal introgression in coastal *A. gambiae*

*Anopheles gambiae* sequences were obtained from 12 individuals from Antula, five from Safim and four from Leibala (Guinea Bissau), and from four additional specimens collected approximately 1,500 km away in Accra, Ghana, a region representative of the low interspecific hybridisation observed in most of the species range (Supplementary Table 3).

**Figure 3:**
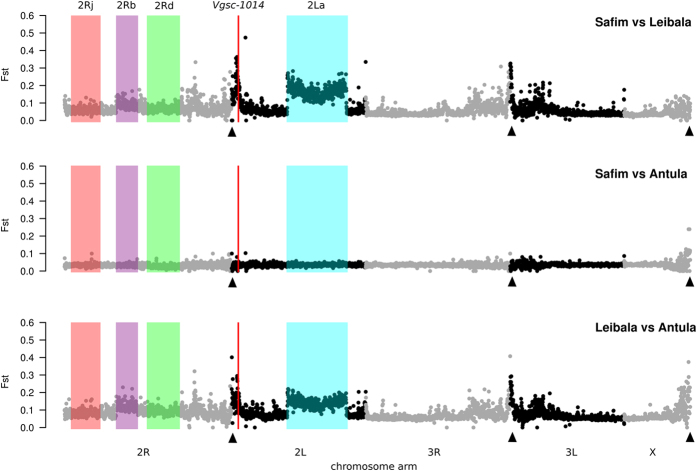
Manhattan plots of mean pairwise *F*_*ST*_ between *Anopheles gambiae* samples of Guinea Bissau. Plots show the three pairwise comparisons of genome-wide differentiation between *A. gambiae* samples from Safim (coastal), Antula (coastal) and Leibala (inland). No *A. coluzzii* individuals were included in the analysis. Arrows indicate the location of the centromeres. The red vertical line indicates position 1014 of the voltage-gated sodium channel gene which corresponds to the *kdr* locus. The four inversion systems scored are also shown by the shaded areas. A 50 kb stepping window was used to generate the mean *F*_*ST*_.

*Anopheles gambiae* coastal samples (Safim *vs.* Antula) showed very low genomic divergence, as expected for a within-species comparison (Fig. 3). However, divergence was elevated in the comparisons between each of these coastal sites and *A. gambiae* from the Leibala inland site. Differentiation (*F*_*ST*_) was especially pronounced at pericentromeric regions on each chromosome, which are known to segregate between *A. coluzzii* and *A. gambiae (e.g.* Neafsey *et al*.^17^), with 22% of *F*_*ST*_ values within the top 5% of genome-wide *F*_*ST*_ values located therein (χ^2^ = 383, *P* = 1 × 10^−85^). Similarly, the 2La inversion region was exceptionally divergent, with 68% of *F*_*ST*_ values in the top 5% (χ^2^ = 1029, *P* = 1 × 10^−226^). However, the other genomic regions corresponding to the karyotypic differences observed among the sampling locations (Fig. 2, Table 2) were not significantly divergent in sequence comparisons (Fig. 3), with no windows in any of the 2R inversion regions located within the top 5% of *F*_*ST*_ values.

**Figure 4:**
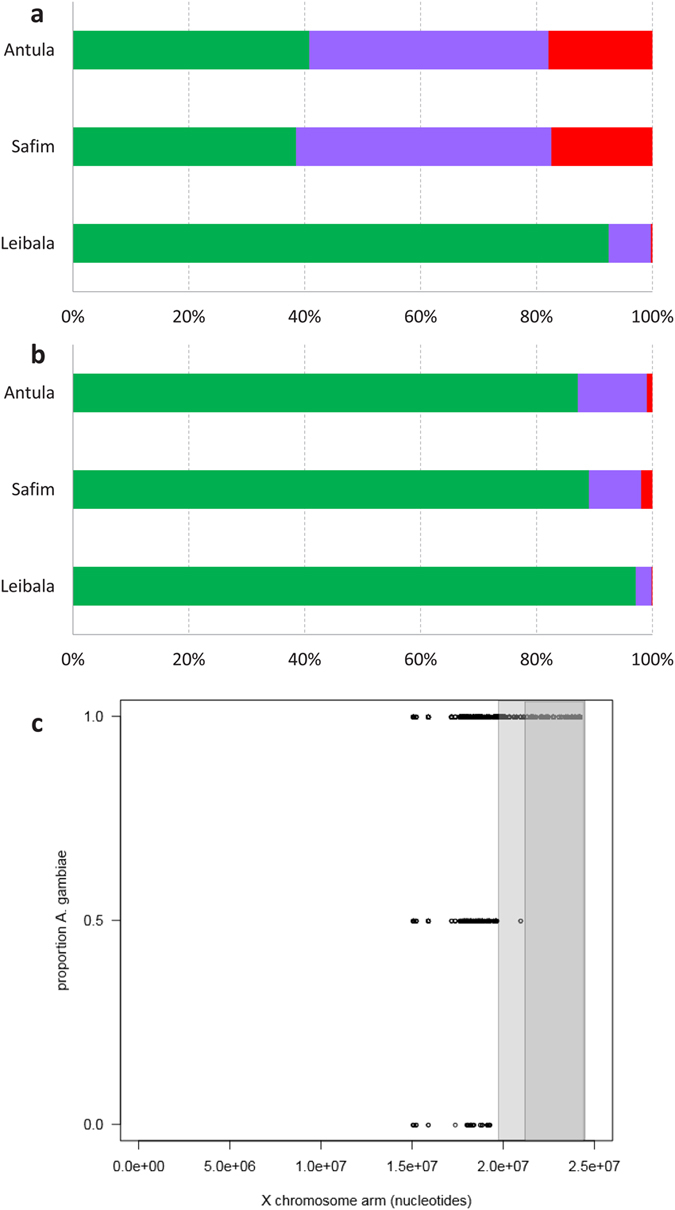
Percentage ancestry based on ancestry informative markers (AIMs) in *Anopheles gambiae* individuals from Guinea Bissau. Stacked bars show percentage of ancestry found in whole genome sequenced individuals sampled in two coastal (Safim *N* = 5, Antula *N* = 12) and one inland locality (Leibala, *N* = 4) estimated using AIMs identified from genome-wide comparisons between samples from a more typical region of infrequent hybridization^17^. Percentage ancestry is based on 93 autosomal (**a**) and 236 chromosome-X (**b**) AIMs, each scored as being homozygous for *A. gambiae* (green) or *A. coluzzii* (red) or heterozygous/admixed (purple). (**c**) Position and genotype of chromosome-X AIMs. Each AIM was genotyped as 0 = *A. coluzzii* homozygote, 0.5 = *A. coluzzii/gambiae* heterozygote or 1 = *A. gambiae* homozygote. Shaded areas correspond to the >3 Mb region (dark grey) homozygous for for *A. gambiae* AIMs, which increases to >4 Mb (light grey) if a single heterozygous AIM is included.

To analyse evidence of introgression from *A. coluzzii* within the samples of *A. gambiae* sequenced, we used Ancestry Informative Markers (AIMs) retrieved from a genome-wide *A. coluzzii vs. A. gambiae* comparison carried out in a region of infrequent hybridization^17^. This yielded 236 Single Nucleotide Polymorphisms (SNPs) on chromosome-X and 93 SNPs on the autosomes in the *A. gambiae* Guinea Bissau samples (Supplementary Fig. 2; see also Supplementary Dataset 1). The proportionate AIM-estimated ancestries for these markers are shown in Fig. 4. Coastal *A. gambiae* from Antula and Safim showed much higher numbers of (putatively introgressed) autosomal *A. coluzzii* AIMs than the inland sample from Leibala (χ^2^ = 333.1, d.f. = 4, *P* < 0.001). Whilst the proportion of *A. coluzzii* AIMs was also higher in coastal sites for chromosome-X (χ^2^ = 88.6, d.f. = 4, *P* < 0.001), this proportion was much lower than on the autosomes (χ^2^ = 124.8, d.f. = 4, *P* < 0.001). Indeed, there was a stretch (>3 Mb) of ‘pure’ *A. gambiae* homozygous AIMs located in the pericentromeric region of chromosome-X (Fig. 4), that was found in all individuals irrespective of sampling location.

**Figure 5:**
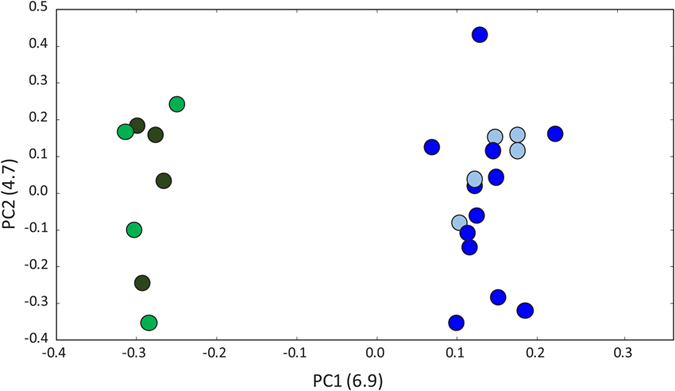
Principal Components Analysis based on chromosome arm 3L variants. Dots represent the relationship between PC1 and PC2 of whole genome sequenced individuals collected in Antula (blue), Safim (light blue), Leibala (green) and Accra-Ghana (dark green).

Genome sequence differentiation between Guinea Bissau and the low hybridisation region of Ghana is shown in the PCA plot of Fig. 5, which was based on the analysis of chromosome-3L SNPs, though 3R SNPs gave a near identical profile (Supplementary Fig. 3). Principal component (PC) 1 explained by far the greatest variance and substitution of alternative secondary principal components did not alter patterns (Supplementary Fig. 3). The PCA clearly separated all coastal Guinea Bissau specimens from a group including the inland Guinea Bissau sample of Leibala and Ghanaian specimens (Fig. 5).

### Medically-relevant loci and traits differ between inland and coastal *A. gambiae*

Two major insecticide resistance loci were genotyped in all specimens that had been analysed using microsatellites. The *Vgsc*-1014F (*kdr*) allele (associated with DDT and pyrethroid resistance) was detected at very low frequency (<5%) in the two central localities of Ga-Mbana and Mandingara but was common in the inland localities, exceeding 95% in Leibala (Fig. 2). This *kdr* allele was entirely absent from all three coastal samples. Among the clusters identified by STRUCTURE, the 1014F allele frequency was 77% in *A. gambiae-*inland but was below 5% in *A. gambiae-*coast and *A. coluzzii* clusters (Table 3). The *ace-1* 119S allele (associated with carbamate and organophosphate resistance) was detected only in inland sites, albeit at low frequencies (Comuda: 2.1%; Leibala: 3.3%) and always as heterozygotes, but was absent elsewhere (Table 3).

Blood-fed females were collected in Safim (*N* = 93) and Antula (*N* = 8) in the coastal region; Mansoa (at the border between coastal and central regions; *N* = 12) and Leibala (inland region; *N* = 30) (Fig. 2). Among STRUCTURE-based genetic clusters, the Human Blood Index (proportionate feeding on humans, HBI) was more than two-fold greater for *A. gambiae*-inland than for *A. gambiae-*coast and *A. coluzzii* (χ^2^ = 24.29, d.f. = 2, *P* < 0.001; Table 3), which exhibited a relatively high frequency of bovine blood meals (30%) (Supplementary Table 4).

## Discussion

We have genetically characterised the species-pair *A. coluzzii* and *A. gambiae* in the zone of extreme hybridization at the far-west of their distribution, in order to investigate the extent of the hybrid zone, possible determinants and consequences of massive introgression for population divergence and malaria transmission and control.

### Hypothesis 1: The hybrid zone is limited to the coastal region and differentiated from inland populations by chromosomal inversions

Results from species diagnostic markers (IGS and SINE) and microsatellites supported the hypothesis that in Guinea Bissau the hybrid zone is confined to the coastal region. Three clusters were detected from the microsatellite data, irrespective of analysis method, comprising of a coastal region dominated by individuals typing as *A. gambiae* or admixed, a central region dominated by *A. coluzzii* and an inland region of *A. gambiae*, suggesting that the core of the hybrid zone is confined to a stretch of no more than 110 km from the coast. Genetic partitioning of coastal and inland *A. gambiae* populations separated by a region dominated by *A. coluzzii* has also been reported along the Gambia river, a few hundred kilometres north of Guinea Bissau^40^.

Based on prior observations from Guinea Bissau^39^, Senegal and The Gambia^34^,^38^ we also proposed that chromosomal inversion polymorphisms, frequently implicated in environmental adaptation^8^,^9^,^10^, would differ between inland and coastal regions. This proved to be the case and we suggest that this is likely to reflect a difference in the frequency of two chromosomal forms. *Anopheles gambiae* at the coast are characterized by a high frequency of the 2Rd inversion and low frequency of 2Rb and 2La, compatible with that described for the BISSAU chromosomal form^8^,^9^ and this inversion pattern was shared with the sympatric *A. coluzzii* population. Inland *A. gambiae* is characterized by higher complexity along chromosome-2R, including the exclusive presence of 2Rj inversion and by high frequency of 2Rb and 2La inversions. This pattern also characterises inland populations from The Gambia^38^,^40^ and is typical of contiguous populations surrounding the Fouta-Djalon massif, which incompletely intergrade with neighbouring populations of the SAVANNA form^9^,^32^,^40^. The concurrence of the BISSAU form and the hybridization zone may not be coincidental but rather linked to the adaptive potential of variation within the 2Rd inversion for exploitation of coastal habitats^8^,^38^,^40^. The alternate frequency of the 2La inversion between the more humid south-western coast (Safim) and the drier north-eastern inland (Leibala) (Supplementary Fig. 4) is in line with previous findings in the region^39^,^40^, and agrees with the well-known latitudinal variation of the 2La inverted karyotype in association with aridity and the savannah-forest transition in West Africa^8^,^10^.

Inland *vs.* coastal differentiation was also clearly evident in genome sequence data for the 2La inversion, which shows large areas of fixed or near fixed differences between orientations^43^. However, no such correspondence of karyotypic and genome sequence variation was evident for the 2Rd inversion region, or others on chromosome arm 2R, suggesting far more sharing of polymorphism between orientations as a result of more recent origin and/or a more diffuse nature of the putatively adaptive variants.

### Hypothesis 2: a distinct hybrid form is being created in the coastal zone

Whilst inland *A. gambiae* is genomically similar to *A. gambiae* from areas of low hybridisation (here represented by the Ghanaian population^5^,^36^), the genomes of coastal *A. gambiae* display signs of extensive introgression from *A. coluzzii*, as shown by the AIM analysis. A similar contrast was also noted in a comparison between samples from distinct inland and coastal localities in Guinea-Bissau, using a 15 SNP panel^19^, indicating that this pattern is not sample site dependent. The single and notable exception to the pattern is the chromosome-X pericentromeric island of interspecific divergence which retains an apparently unbroken *A. gambiae* haplotype >3 Mb long. While the pattern of autosomal introgression from *A. coluzzii* into *A. gambiae* agrees with the asymmetric introgression pattern demonstrated in the coastal region^7^, AIM analysis showed that introgression can only partially penetrate chromosome-X. Although sequence data comes from a small number of *A. gambiae* specimens selected in advance for consistency between IGS and SINE, this observation coupled with proximity to the centromere agree with a lower recombination and/or stronger selection against recombinants at the X-pericentromeric island as compared to the rest of the genome. This is concordant with results from a study genotyping hemizygous males from the same geographic region^37^, which detected limited recombination in the X-pericentromeric island.

Given the geographical zonation of species detected, opportunities for introgressive backcrossing of the coastal admixed population appear to be diminishing. Therefore, rather than a putative decline of *A. coluzzii* or progressive erosion of divergence by introgression in the coastal region, what we appear to be observing is creation of a novel hybrid form by strong asymmetric introgression, characterised by an admixed genome and an *A. gambiae* like X-pericentromeric island. Merging of the *A. coluzzii* genome by introgression into *A. gambiae* but with an apparently highly conserved stretch of *A. gambiae* genome on the heterogametic sex chromosome – which has recently been implicated in partial reproductive isolation between the species^21^ – represents an extraordinary example of genomic flexibility, which may be characteristic of the species complex^14^. Further temporal monitoring is required to examine the stability of divergence of this putative hybrid form and the extent to which fluctuations, *e.g.* related to seasonality, may affect its occurrence.

The finding of a hybrid form typing as *A. gambiae* by species-specific markers located in the X-pericentromeric island raises the question of whether this form might also be found ‘hidden’ elsewhere throughout the sympatric range of the species-pair. This may be true but probably to a limited extent because: (i) the stable elevated hybrid rates reported in the “far-west” African region have never been reported outside this region; (ii) although available genome-wide data remains geographically sparse, in low hybridization regions additional species diagnostic markers on chromosome-3L are often found in linkage disequilibrium with those on chromosome-X^19^; (iii) coastal areas, which represent a relatively small fraction of the range of the species-pair, may have peculiar characteristics favouring the evolution of a new form better adapted to elevated breeding site salinity to which *A. coluzzii*, and especially *A. gambiae*, have limited tolerance, by comparison to the brackish water-breeding west African species *A. melas*^29^. Nevertheless, given that recent introgression is detectable even in low hybridization countries^19^, genomic examination of coast-to-inland transects from elsewhere would certainly be of interest.

The timing and mechanisms underlying the breakage in reproductive isolation between *A. coluzzii* and *A. gambiae* require further investigation. However, it is interesting to note that environmental changes associated with the implementation of rice cultivation occurred in Guinea Bissau within a relatively recent time scale. Mangrove swamp rice cultivation was established in the 15^th^ century by farmers of the Balanta ethnic group in the central part of the Mansoa river valley and subsequently expanded westwards to the coast along the major rivers of the country^44^. The establishment of rice fields in coastal areas could have provided the means for *A. coluzzii* – known to be better adapted to agricultural larval habitats^26^ and slightly more salinity tolerant than *A. gambiae*^30^ – to expand its range into the salt-water mangrove swamp coastal areas of Guinea Bissau. A recent expansion (*i.e.* 500 year ago) could have promoted contact between *A. coluzzii* and an already-present coastal *A. gambiae* population, providing the opportunity for the establishment of a secondary contact zone. This would present concomitant potential for introgression to create novelty for adaptation to the coastal region. A similar pattern of *A. coluzzii* predominance in rice-cultivated central regions and increased hybridisation westwards towards the coast was also found in The Gambia^40^. In one of the brackish water-breeding specialists, the east African species *A. merus*, salinity tolerance maps to multiple QTL including the large 2Rop inversion region^45^, which encompasses the 2Rd inversion in *A. coluzzii* and *A. gambiae*. Though speculative at present, involvement of the 2Rd inversion in salinity tolerance is consistent with its observed distribution and may be a key adaptation in more coastally adapted *A. coluzzii*^30^. The extent to which anthropogenic activity may have influenced the establishment of a secondary contact zone and introgression is unclear, but introgression between closely related taxa associated with human-mediated environmental change has been described for a number of other organisms^46^, and can affect not only the genetic integrity of species but also their capacity to adapt to altered environments^3^.

### Hypothesis 3: Establishment of a hybrid form may promote aggregation of medically important mutations and phenotypes

Our hypothesis that hybridisation might influence traits related to malaria transmission and control through aggregation of medically important mutations and phenotypes was not met for the traits investigated here (*i.e.* insecticide resistance associated mutations and host preference). Previously we found an elevated frequency of an introgressed *A. coluzzii* specific *tep1r1 Plasmodium*-resistant allele in coastal *A. gambiae*^47^. Whilst evidence for an increased zoophilic tendency detected in *A. gambiae* coupled with the introgression of the *tep1r1* parasite-resistant allele might predict a lower contribution of this species to malaria transmission in the coastal area, *P. falciparum* infection rates were actually higher in *A. gambiae* than in sympatric *A. coluzzii* collected in coastal Guinea Bissau in 2009^42^. Rather than hybridization, it is the partitioning of *A. gambiae* into two genetically distinct subpopulations that currently appears to have public health implications. This genetic partitioning into coastal and inland populations probably involves restrictions to gene flow arising from physical isolation by the central region (represented by Mansoa, Mandigara and Ga-Mbana) where *A. coluzzii* predominates. There was evidence for marked differences between coastal and inland *A. gambiae* subpopulations in both host preference and in the frequency of the *kdr* insecticide resistance allele.

The contrasting *kdr* frequencies between coast and inland subpopulations could also have implications for vector control. Different insecticide selective pressures could hypothetically explain this observation but there is no evidence of higher insecticide pressure in inland Guinea Bissau. Insecticide treated nets are the only anti-vector measure used by the malaria control programme of Guinea Bissau^48^ and these would exert a highest pressure in coastal areas, where most population of the country lives. There have also been reports associating *kdr* resistance with insecticide usage in cotton crops^49^. However, cotton is not among the most important produces of Guinea Bissau^50^. A more plausible explanation is that inland *A. gambiae* is part of the larger western African *A. gambiae* population known to display high *kdr* frequency^22^, which agrees with the grouping of this population with individuals from Ghana in the whole genome sequence PCA.

## Conclusion

Genomic introgression, coupled with restrictions to gene flow due to habitat segregation in the central region of the country, are promoting divergence between two genetically distinct *A. gambiae* populations in Guinea Bissau. One is a coastal, *A. coluzzii-*introgressed population, characterized by lower inversion polymorphism (excepting the 2Rd inversion), greater zoophilic tendency and absence of *kdr* mutations; the other is an inland, more typical, anthropophilic *A. gambiae* population potentially more adapted to aridity and displaying high *kdr* frequency. In other hybrid zones, asymmetrical introgression is often driven by mate choice (see While *et al*.^51^ and references therein). At present in the Guinea Bissau hybrid zone the drivers of this asymmetry are not clear, and further investigation is now warranted. Epidemiological studies are required to determine how this zonation is impacting malaria transmission. Monitoring of the stability of the genetic partitioning is also crucial, both from the perspective of providing a window into the timescale of what may represent contemporary adaptive introgression, perhaps on a trajectory towards hybrid speciation, and for the potential that disruption of the current partitioning could lead to a salinity tolerant, insecticide resistant hybrid form with a higher capacity to transmit malaria in the densely-populated coastal region of Guinea Bissau and neighbouring west African countries.

## Methods

### Mosquito sampling

Mosquito collections took place between 10^th^ and 30^th^ October 2010 in eight localities along a 180 km southwest-northeast transect covering three major regions of Guinea Bissau (Fig. 2, Supplementary Fig. 4): (i) a south-western coastal region, characterised mainly by mixed flooded forests and croplands and with a mean aridity index >1.0 (localities of Quinhamel, Safim and Antula); (ii) a central region, where large patches of evergreen forest are present (Mansoa, Mandingara and Ga-Mbana) and mean aridity index between 0.8 and 1.0; (iii) a north-eastern inland region where shrubland and open deciduous forest appear to prevail and with a mean aridity index between 0.6 and 0.8 (Comuda and Leibala).

Host-seeking mosquitoes were collected indoors (19:00–07:00) by CDC miniature light traps. These mosquitoes were stored individually in tubes filled with silica gel and cotton for DNA analyses. In addition, indoor resting collections were performed. The midgut contents of blood fed females caught resting were squashed onto filter paper (Whatman^®^ Grade 1) for blood meal analysis. The ovaries of half-gravid females were preserved in Carnoy’s fixative (absolute ethanol: glacial acetic acid, 3:1) and stored at −20 °C for polytene chromosome preparations. The remainder of the carcasses of half-gravid and blood fed females was also kept dry in silica gel until DNA extraction.

### Molecular identification

DNA extraction from individual females was performed using a phenol: chloroform protocol^52^. Molecular identification of species was performed by PCR-RFLP targeting species-specific polymorphisms at the intergenic spacer (IGS) of the ribosomal DNA^53^ and by a PCR assay targeting the SINE 200X6.1 retrotransposon insertion^31^. Specimens were identified as *A. coluzzii* or *A. gambiae* if they had coincident species-specific patterns for both markers. Specimens exhibiting either a consistent *A. coluzzii/A. gambiae* heterozygous pattern for both IGS and SINE or a discordant result between markers were classified as admixed.

### Microsatellite genotyping

Nineteen microsatellites, nine mapping on chromosome-X and ten on chromosome-3, were genotyped (Supplementary Table 1). Each locus was PCR amplified with fluorescently labelled primers^52^ and fragment analysis was performed on an automated sequencer at the Science Hill DNA Analysis Facility, Yale University. To control for variation between capillary runs, the PCR products of two *A. coluzzii* (SUAKOKO strain) specimens from a laboratory colony were used in all runs. One additional positive control (DNA template from a colony mosquito) and one negative control (no template) were also included to assess PCR quality. Allele sizes were scored using GENEMARKER^®^ (SoftGenetics, PA, USA).

Genetic diversity was assessed by estimates of allele richness (*A*_*R*_), expected heterozygosity (*H*_*e*_) and inbreeding coefficient (*F*_*IS*_) available in FSTAT v. 2.9.3.2^54^. Departures from Hardy-Weinberg proportions were tested by exact tests using ARLEQUIN v.3.5^55^. Presence of null alleles was tested using MICRO-CHECKER with the complete range option^56^. Whenever multiple tests were performed the nominal significance level (α = 0.05) was adjusted by a sequential Bonferroni procedure.

The Bayesian clustering analysis method implemented in STRUCTURE 2.3.3^57^ was used to infer the number of genetic clusters (*K*) without prior information of sampling locations. A model with correlated allele frequencies within populations (*λ* = 1) with the option of admixture (α was allowed to vary) was used. For each value of *K (K* = 1 to 10) 10 independent runs were performed with a burn-in period of 100,000 iterations followed by 200,000 iterations. Two *ad hoc* approaches implemented in Structure Harvester v.0.6.94^58^ were used to determine *K*: (i) an estimation of ln[Pr(X|*K*)]^57^; and ii) the *ΔK* statistic^59^. Upon selecting *K*, data across runs were optimally aligned with CLUMPP^60^ using the Greedy algorithm. Assignment of individual mosquitoes in to genetic clusters was performed based on a probability threshold (*T*_*q*_) of 0.50.

Spatially-explicit genetic clustering analysis was conducted using TESS v.2.3^61^. Individual coordinates for each specimen were randomly generated within a 10 km radius circle around the geographic coordinate of each locality. The two admixture models (CAR and BYM) were used in the analysis. Ten independent runs were carried out with a burn-in period of 100,000 iterations and 100,000 data-collection iterations for each *K*_*max*_ (*K* = 2 to *K* = 9). The Deviance Information Criterion (DIC) was used to select and to infer the number of clusters. The best performing admixture model and optimal *K*_*max*_ were selected from plots of the DIC and *K*_*max*_ as the lowest value at which the DIC curve reached a plateau. Individual membership probabilities of the ten runs for the optimal *K*_*max*_ were averaged using the greedy algorithm in CLUMPP. The R-script available in POPS^62^ was used to display spatial interpolations of the Q matrices obtained with TESS based on kriging methods.

### Chromosomal analysis

Polytene chromosome preparations were obtained from the ovaries of half-gravid females and banding patterns were scored under a phase-contrast microscope (400X magnitude). Paracentric inversion karyotypes were named as follows: standard (non-inverted, *i.e.* 2R+^^j^^, +^^b^^, +^^d^^ and 2L+^^a^^) and inverted arrangements (2Rj, b, d and 2La) were considered as alternative alleles of a bi-allelic locus (where locus = chromosomal region containing an inversion polymorphism and allele = inversion orientation arrangement). BISSAU and SAVANNA chromosomal forms were defined according to the criteria of Bryan *et al*.^38^, Coluzzi *et al*.^8^ and Petrarca *et al*.^39^.

### Whole genome sequencing

Mosquitoes identified as *A. gambiae* by IGS/SINE from three sites in Guinea Bissau were sequenced using Illumina^®^ technology at the Wellcome Trust Sanger Institute (Hinxton, UK). Individuals from Safim and Leibala were sequenced specifically for this study. Genomic DNA library preparation (input DNA amount >50 ng), cluster generation and sequencing were undertaken according to the manufacturer’s protocol for paired-end 100 bp sequence reads. In addition, a sample from Antula was retrieved from the genomes available at the *Anopheles gambiae 1000 genomes* consortium^63^. Additional *A. gambiae* and also *A. coluzzii* specimens were sequenced but did not yield sufficient quality sequence for inclusion after conservative filtering (see below). Finally, *A. gambiae* genomes from Accra, Ghana, were taken from Clarkson *et al*.^5^.

To ensure that all samples were comparable and that there was high confidence in the variants scored, raw SNPs called using the Unified Genotyper algorithm from the GATK package^64^ were conservatively hard filtered using a custom Python script. Unlike simple ‘hard filtering’, the script allowed the DP annotation distribution to be considered for each individual and variants were rejected if they possessed the following annotation parameters: GQ < 40, DP < 14, DP < median DP/2 or DP > median DP x 2, MQ < 40, QD < 5, HRun > 3. The final two filtering rules were taken from the Human 1000 Genomes Project^65^. The additional rules were trialled against an unpublished data set of *A. gambiae* laboratory crosses and were found to reduce Mendelian errors compared to just the human filtering rules.

To further increase the quality of these datasets, positions with missing genotypes in any sample were removed using VCFtools package (v0.1.12a)^66^. In analyses where only Guinea Bissau sequences were compared, all filtered SNP calls were merged and positions missing data in any individual were removed using VCFtools to create a Guinea Bissau-missingless VCF file. For analyses which included samples from Ghana, the four additional individuals’ VCF files were merged with the Guinea Bissau-missingless VCF (using VCFtools) and missing sites were removed again to produce an all-sample-missingless VCF file. This was carried out to preserve the high resolution of variants in the Guinea Bissau-only analyses owing to the higher sequencing depth of those samples. Guinea Bissau samples had a mean coverage of 32.3x compared to 23.8x for Ghanaian samples (Supplementary Table S3).

Pairwise population *F*_*ST*_ estimates were calculated using *weir-fst-pop* available in VCFtools. *F*_*ST*_ means, standard errors and 95% confidence intervals were then calculated using custom Perl and R scripts and Manhattan plots were created with the R package qqman^67^.

As *A. coluzzii* individual whole genome sequence data were not available, an ancestry informative marker (AIM) approach was used^5^,^19^,^20^. Informative markers were obtained from a 400k SNP chip used to characterise divergence between Malian *A. gambiae* and *A. coluzzii*^17^. SNPs with allele frequency differences >0.9 between species were selected as being ancestry informative. Percentage ancestry was assessed by scoring all Guinea Bissau individuals at each marker as being of homozygous *A. gambiae*, homozygous *A. coluzzii* or heterozygous (admixed) ancestry. Autosomes and chromosome-X were examined separately because chromosome-X has been shown to be less susceptible to introgression^14^ and because it has a higher number of AIMs than all the autosomes combined^5^,^15^.

To produce a maximally informative dataset for principal component analysis (PCA), singletons were removed and linkage disequilibrium (LD) pruning was conducted using 500 SNP windows, sliding 100 SNP at a time, with an LD (*r*^*2*^) threshold of 0.1. A low *r*^*2*^ threshold was used due to the high genetic diversity and low linkage disequilibrium found in these mosquito species^17^. Calculations were done using the all-sample-missingless VCF and the software PLINK (v1.9)^68^. PCA was then performed on the 3L and 3R chromosome arms using the *smartpca* function in the EIGENSOFT (6.0.1) package^69^. Chromosome-3 was chosen to better reflect relation between samples with respect to gene flow, because this chromosome is less affected by confounding factors such as large polymorphic inversions (*i.e.* such as those found on chromosome arms 2L and 2R) and regions related to speciation (*i.e.* such as the centromeric region on chromosome*-*X)^14^.

#### Insecticide resistance genes

Position 1014 at the voltage-gated sodium channel gene (VGSC^1014^), in which two mutations (L1014F^70^ and L1014S^71^) are associated with knockdown resistance (*kdr*), was genotyped by PIRA-PCR^72^. The G119S mutation in the acetylcholinesterase-1 (*ace*-1) gene associated with resistance to carbamates and organophosphates was genotyped by PCR-RFLP^73^.

### Blood meal identification

A two-site ELISA^74^ was used to identify the origin of blood meal in engorged females. Blood meals were tested for the presence of chicken, cow, dog, goat/sheep, horse/donkey, human, pig, and rabbit immunoglobulin G (IgG). Four positive controls (blood from the tested host) and 14 negative controls (two blood samples from the other seven hosts) were used in every 96-well microplate. Absorbance was read at 492 nm wave length and cut-off values were calculated for each plate as the mean plus three times the standard deviation of the negative controls.

## Additional Information

**How to cite this article:** Vicente, J. L. *et al*. Massive introgression drives species radiation at the range limit of *Anopheles gambiae. Sci. Rep.* **7**, 46451; doi: 10.1038/srep46451 (2017).

**Publisher's note:** Springer Nature remains neutral with regard to jurisdictional claims in published maps and institutional affiliations.

## Supplementary Material

Supplementary Figures and Tables

Supplementary Dataset 1

## Figures and Tables

**Table 1:** Association between Bayesian genetic clusters (STRUCTURE and TESS) and molecular identification of species by IGS and SINE.

		Structure				Tess			
		Cluster 1	Cluster 2	Cluster 3	Admixed	Cluster 1	Cluster 2	Cluster 3	Admixed
IGS/SINE	*A. coluzzii*	**0.882**	0.022	0.022	0.104	**0.914**	0.006	0.026	0.111
	Admixed	0.062	0.062	0.398	**0.463**	0.062	0.000	0.436	0.111
	*A. gambiae*	0.057	**0.916**	**0.580**	0.433	0.024	**0.994**	**0.538**	**0.778**
	*N*	211	178	231	67	210	163	305	9

Values represent the relative proportions of each species (determined by IGS/SINE markers) within each STRUCTURE or TESS cluster. Highest proportions are highlighted in bold. *N*: total number of specimens assigned to each cluster based on a probability threshold (*T*_*q*_) of 0.50.

**Table 2:** Karyotype frequencies (%) in Safim (coast) and Leibala (inland), Guinea Bissau.

Chromosome-2R karyotypes	Safim			Leibala
	*A. coluzzii*	Admixed	*A. gambiae*	*A. gambiae*
+/+		25.0	23.7	6.9
+/d	66.7	56.6	51.8	8.6
d/d	16.7	17.1	22.8	
+/b				29.3
b/b				17.2
bd/+, b/d	16.7		0.9	8.6
jb/+, j/b				10.3
jbd/+, j/bd, jb/d				6.9
jb/b				3.4
jbd/b, jb/bd				5.2
jb/d, jd/b, j/bd				3.4
bd/d			0.9	
d/u		1.3		
Total	12	76	114	58
Chromosome-2L karyotypes				
+/+	66.7	61.8	61.4	5.2
+/a	33.3	32.9	36.8	32.8
a/a		5.3	1.8	62.1
Total	12	76	114	58

**Table 3:** Distribution of insecticide resistance-associated alleles and human blood index among Bayesian genetic clusters (STRUCTURE).

	Cluster 1 (*A. coluzzii*)	Cluster 2 (*A. gambiae-*inland)	Cluster 3 (*A. gambiae-*coast)	Admixed
*kdr f*_(1014F)_	0.019	0.767	0.045	0.179
	[0.009–0.039]	[0.714–0.809]	[0.029–0.070]	[0.120–0.257]
	(211)	(176)	(231)	(67)
*ace-1 f*_(119S)_	0.002	0.014	0.000	0.022
	[0.000–0.015]	[0.005–0.034]	[0.000–0.010]	[0.006–0.069]
	(211)	(178)	(231)	(67)
HBI	0.385	0.824	0.308	0.391
	[0.151–0.677]	[0.648–0.926]	[0.202–0.435]	[0.205–0.612]
	(13)	(34)	(65)	(23)

Values represent proportions and 95% confidence intervals in square brackets of the resistance-associated alleles in the case of *kdr* and *ace-1* and blood meals taken from humans, in the case of the human blood index (HBI), respectively. Sample sizes are in parenthesis.

## References

[b1] Kleindorfer S. . Species collapse via hybridization in Darwin’s tree finches. Am. Nat. 183, 325–341 (2014). 24561597 10.1086/674899

[b2] Roy D., Lucek K., Walter R. P. & Seehausen O. Hybrid ‘superswarm’ leads to rapid divergence and establishment of populations during a biological invasion. Mol. Ecol. 24, 5394–5411 (2015). 26426979 10.1111/mec.13405

[b3] Nolte A. W. & Tautz D. Understanding the onset of hybrid speciation. Trends Genet. 26, 54–58 (2010). 20044166 10.1016/j.tig.2009.12.001

[b4] Pardo-Diaz C. . Adaptive introgression across species boundaries in *Heliconius* butterflies. PLoS Genet. 8, e1002752, doi: 10.1371/journal.pgen.1002 (2012). 22737081 PMC3380824

[b5] Clarkson C. S. . Adaptive introgression between *Anopheles* sibling species eliminates a major genomic island but not reproductive isolation. Nat. Commun. 5, 4248, doi: 10.1038/ncomms5248 (2014). 24963649 PMC4086683

[b6] Oliveira E. . High levels of hybridization between molecular forms of *Anopheles gambiae* from Guinea Bissau. J. Med. Entomol. 45, 1057–1063 (2008). 19058629 10.1603/0022-2585(2008)45[1057:hlohbm]2.0.co;2

[b7] Marsden C. D. . Asymmetric introgression between the M and S forms of the malaria vector, *Anopheles gambiae*, maintains divergence despite extensive hybridization. Mol. Ecol. 20, 4983–4994 (2011). 22059383 10.1111/j.1365-294X.2011.05339.x PMC3222736

[b8] Coluzzi M., Petrarca V. & Di Deco M. A. Chromosomal inversion intergradation and incipient speciation in *Anopheles gambiae*. Boll. Zool. 52, 45–63 (1985).

[b9] Coluzzi M., Sabatini A., della Torre A., di Deco M. A. & Petrarca V. A polytene chromosome analysis of the *Anopheles gambiae* species complex. Science 298, 1415–1418 (2002). 12364623 10.1126/science.1077769

[b10] Ayala D., Ullastres A. & González J. Adaptation through chromosomal inversions in *Anopheles*. Front. Genet. 5, 129, doi: 10.3389/fgene.2014.00129 (2014). 24904633 PMC4033225

[b11] della Torre A. . Molecular evidence of incipient speciation within *Anopheles gambiae* s.s. in West Africa. Insect Mol. Biol. 10, 9–18 (2001). 11240632 10.1046/j.1365-2583.2001.00235.x

[b12] Coetzee M. . *Anopheles coluzzii* and *Anopheles amharicus*, new members of the *Anopheles gambiae* complex. Zootaxa 3619, 246–724 (2013). 26131476

[b13] Feder J. L., Egan S. P. & Nosil P. The genomics of speciation-with-gene-flow. Trends Genet. 28, 342–350 (2012). 22520730 10.1016/j.tig.2012.03.009

[b14] Fontaine M. C. . Extensive introgression in a malaria vector species complex revealed by phylogenomics. Science 347, 1258524 (2015). 25431491 10.1126/science.1258524 PMC4380269

[b15] Turner T. L., Hahn M. W. & Nuzhdin S. V. Genomic islands of speciation in *Anopheles gambiae*. PLoS Biol. 3, e285, doi: 10.1371/journal.pbio.0030285 (2005). 16076241 PMC1182689

[b16] White B. J., Cheng C., Simard F., Costantini C. & Besansky N. J. Genetic association of physically unlinked islands of genomic divergence in incipient species of *Anopheles gambiae*. Mol. Ecol. 19, 925–939 (2010). 20149091 10.1111/j.1365-294X.2010.04531.x PMC3683534

[b17] Neafsey D. E. . SNP genotyping defines complex gene-flow boundaries among African malaria vector mosquitoes. Science 330, 514–517 (2010). 20966254 10.1126/science.1193036 PMC4811326

[b18] Cruickshank T. E. & Hahn M. W. Reanalysis suggests that genomic islands of speciation are due to reduced diversity, not reduced gene flow. Mol. Ecol. 23, 3133–3157 (2014). 24845075 10.1111/mec.12796

[b19] Lee Y. . Spatiotemporal dynamics of gene flow and hybrid fitness between the M and S forms of the malaria mosquito, *Anopheles gambiae*. Proc. Natl Acad. Sci. USA 110, 19854–19859 (2013). 24248386 10.1073/pnas.1316851110 PMC3856788

[b20] Norris L. C. . Adaptive introgression in an African malaria mosquito coincident with the increased usage of insecticide-treated bed nets. Proc. Natl. Acad. Sci. USA 112, 815–820 (2015). 25561525 10.1073/pnas.1418892112 PMC4311837

[b21] Aboagye-Antwi F. . Experimental swap of *Anopheles gambiae*’s assortative mating preferences demonstrates key role of X-chromosome divergence island in incipient sympatric speciation. PLoS Genet. 11, e1005141, doi: 10.1371/journal.pgen.1005141 (2015). 25880677 PMC4400153

[b22] Santolamazza F. . Distribution of knock-down resistance mutations in *Anopheles gambiae* molecular forms in west and west-central Africa. Malar. J. 7, 74, doi: 10.1186/1475-2875-7-74 (2008). 18445265 PMC2405802

[b23] Main B. J. . Complex genome evolution in *Anopheles coluzzii* associated with increased insecticide usage in Mali. Mol Ecol. 24, 5145–5157 (2015). 26359110 10.1111/mec.13382 PMC4615556

[b24] Opondo K. O. . Does insecticide resistance contribute to heterogeneities in malaria transmission in The Gambia? Malar J. 15, 166, doi: 10.1186/s12936-016-1203-z (2016). 26980461 PMC4793517

[b25] White B. J. . Adaptive divergence between incipient species of *Anopheles gambiae* increases resistance to Plasmodium. Proc. Natl Acad. Sci. USA 108, 244–249 (2011). 21173248 10.1073/pnas.1013648108 PMC3017163

[b26] Diabate A. . Evidence for divergent selection between the molecular forms of *Anopheles gambiae*: role of predation. BMC Evol. Biol. 8, 5, doi: 10.1186/1471-2148-8-5 (2008). 18190719 PMC2217532

[b27] Diabate A., Dabire R. K., Millogo N. & Lehmann T. Evaluating the effect of postmating isolation between molecular forms of *Anopheles gambiae* (Diptera: Culicidae). J. Med. Entomol. 44, 60–64 (2007). 17294921 10.1603/0022-2585(2007)44[60:eteopi]2.0.co;2

[b28] Lehmann T. & Diabaté A. The molecular forms of *Anopheles gambiae*: A phenotypic perspective. Infect. Genet. Evol. 8, 737–746 (2008). 18640289 10.1016/j.meegid.2008.06.003 PMC2731232

[b29] Kamdem C. . Anthropogenic habitat disturbance and ecological divergence between incipient species of the malaria mosquito *Anopheles gambiae*. PLoS ONE 7, e39453, doi: 10.1371/journal.pone.0039453 (2012). 22745756 PMC3382172

[b30] Tene Fossog B. . Habitat segregation and ecological character displacement in cryptic African malaria mosquitoes. Evol. Appl. 8, 326–345 (2015). 25926878 10.1111/eva.12242 PMC4408144

[b31] Santolamazza F. . Insertion polymorphisms of SINE200 retrotransposons within speciation islands of *Anopheles gambiae* molecular forms. Malar. J. 7, 163, doi: 10.1186/1475-2875-7-163 (2008). 18724871 PMC2546427

[b32] della Torre A., Tu Z. J. & Petrarca V. On the distribution and genetic differentiation of *Anopheles gambiae* s.s. molecular forms. Insect Biochem. Mol. Biol. 35, 755–769 (2005). 15894192 10.1016/j.ibmb.2005.02.006

[b33] Caputo B. . *Anopheles gambiae* complex along The Gambia river, with particular reference to the molecular forms of *An. gambiae* s.s. Malar. J. 7, 182, doi: 10.1186/1475-2875-7-182 (2008). 18803885 PMC2569043

[b34] Gordicho V. . First report of an exophilic *Anopheles arabiensis* population in Bissau City, Guinea-Bissau: recent introduction or sampling bias? Malar. J. 13, 423, doi: 10.1186/1475-2875-13-423 (2014). 25370807 PMC4240859

[b35] Caputo B. . The “far-west” of *Anopheles gambiae* molecular forms. PLoS One 6, e16415, doi: 10.1371/journal.pone.0016415 (2011). 21347223 PMC3039643

[b36] Weetman D., Wilding C. S., Steen K., Pinto J. & Donnelly M. J. Gene flow-dependent genomic divergence between *Anopheles gambiae* M and S Forms. Mol. Biol. Evol. 29, 279–291 (2012). 21836185 10.1093/molbev/msr199 PMC3259608

[b37] Caputo B. . The last bastion? X chromosome genotyping of *Anopheles gambiae* species pair males from a hybrid zone reveals complex recombination within the major candidate ‘genomic island of speciation’. Mol. Ecol. 25, 5719–5731 (2016). 27661465 10.1111/mec.13840

[b38] Bryan J. H., di Deco M. A., Petrarca V. & Coluzzi M. Inversion polymorphism and incipient speciation in *Anopheles gambiae* s.str. in The Gambia, West Africa. Genetica 59, 167–176 (1982).

[b39] Petrarca V., Carrara G. C., Di Deco M. A. & Petrangeli G. The *Anopheles gambiae* complex in Guinea Bissau. Parassitologia 25, 29–39 (1983). 6543935

[b40] Caputo B. . Prominent intraspecific genetic divergence within *Anopheles gambiae* sibling species triggered by habitat discontinuities across a riverine landscape. Mol. Ecol. 23, 4574–4589 (2014). 25040079 10.1111/mec.12866

[b41] Nwakanma D. C. . Breakdown in the process of incipient speciation in *Anopheles gambiae*. Genetics 193, 1221–1231 (2013). 23335339 10.1534/genetics.112.148718 PMC3606099

[b42] Sanford M. R. . *Plasmodium falciparum* infection rates for some *Anopheles* spp. from Guinea-Bissau, West Africa [version 2; referees: 2 approved]. F1000Research 3, 243, doi: 10.12688/f1000research.5485.2 (2014). 25383188 PMC4215749

[b43] White B. J. . The population genomics of trans-specific inversion polymorphisms in *Anopheles gambiae*. Genetics 183, 275–288 (2009). 19581444 10.1534/genetics.109.105817 PMC2746151

[b44] Kyle S. Rice sector policy options in Guinea Bissau. *Working paper* (Charles H Dyson School of Applied Economics and Management, Cornell University, Ithaca, New York (2015).

[b45] Smith H. A. . Genome-wide QTL mapping of saltwater tolerance in sibling species of *Anopheles* (malaria vector) mosquitoes. Heredity 115, 471–479 (2015). 25920668 10.1038/hdy.2015.39 PMC4611235

[b46] Crispo E., Moore J. S., Lee-Yaw J. A., Gray S. M. & Haller B. C. Broken barriers: human-induced changes to gene flow and introgression in animals: an examination of the ways in which humans increase genetic exchange among populations and species and the consequences for biodiversity. Bioessays 33, 508–518 (2011). 21523794 10.1002/bies.201000154

[b47] Mancini E. . Adaptive potential of hybridization among malaria vectors: introgression at the immune locus TEP1 between *Anopheles coluzzii* and *A. gambiae* in ‘Far-West’ Africa. PLoS One 10, e0127804, journal.pone.0127804 (2015). 26047479 10.1371/journal.pone.0127804 PMC4457524

[b48] WHO. *World Malaria Report, 2014* (World Health Organization, Geneva, 2014).

[b49] Yadouleton A. . Cotton pest management practices and the selection of pyrethroid resistance in *Anopheles gambiae* population in northern Benin. Parasit. Vectors 4, 60, doi: 10.1186/1756-3305-4-60 (2011). 21489266 PMC3082239

[b50] FAO. Guinea Bissau country profile: Major food and agriculture productions. http://faostat.fao.org/DesktopDefault.aspx?PageID=339&lang=en&country=175 (Date of assess: 10/05/2016) (2012).

[b51] While G. M. . Sexual selection drives asymmetric introgression in wall lizards. Ecol. Lett. 18, 1366–1375 (2015). 26468006 10.1111/ele.12531

[b52] Donnelly M. J., Cuamba N., Charlwood J. D., Collins F. H. & Townson H. Population structure in the malaria vector, *Anopheles arabiensis* Patton, in East Africa. Heredity 83, 408–417 (1999). 10583542 10.1038/sj.hdy.6885930

[b53] Fanello C., Santolamazza F. & della Torre A. Simultaneous identification of species and molecular forms of the *Anopheles gambiae* complex by PCR-RFLP. Med. Vet. Entomol. 16, 461–464 (2002). 12510902 10.1046/j.1365-2915.2002.00393.x

[b54] Goudet J. FSTAT (Version 1.2): A computer program to calculate F-statistics. J. Hered. 86, 485–486 (1995).

[b55] Excoffier L., Laval G. & Schneider S. Arlequin (version 3.0): An integrated software package for population genetics data analysis. Evol. Bioinf. Online 1, 47–50 (2005). PMC2658868 19325852

[b56] Van Oosterhout C., Hutchinson W. F., Wills D. P. M. & Shipley P. MICRO-CHECKER: software for identifying and correcting genotyping errors in microsatellite data. Mol. Ecol. Notes 4, 535–538 (2004).

[b57] Pritchard J. K., Stephens M. & Donnelly P. Inference of population structure using multilocus genotype data. Genetics 155, 945–959 (2000). 10835412 10.1093/genetics/155.2.945 PMC1461096

[b58] Earl D. A. & VonHoldt B. M. Structure Harvester: A website and program for visualizing STRUCTURE output and implementing the Evanno method. Conserv. Genet. Resour. 4, 359–361 (2012).

[b59] Evanno G., Regnaut S. & Goudet J. Detecting the number of clusters of individuals using the software STRUCTURE: a simulation study. Mol. Ecol. 14, 2611–2620 (2005). 15969739 10.1111/j.1365-294X.2005.02553.x

[b60] Jakobsson M. & Rosenberg N. CLUMPP: a cluster matching and permutation program for dealing with label switching and multimodality in analysis of population structure. Bioinformatics 23, 1801–1806 (2007). 17485429 10.1093/bioinformatics/btm233

[b61] Chen C., Durand E., Forbes F. & Francois O. Bayesian clustering algorithms ascertaining spatial population structure: a new computer program and a comparison study. Mol. Ecol. Notes 7, 747–756 (2007).

[b62] Jay F. . Forecasting changes in population genetic structure of alpine plants in response to global warming. Mol. Ecol. 21, 2354–2368 (2012). 22512785 10.1111/j.1365-294X.2012.05541.x

[b63] The *Anopheles gambiae* 1000 Genomes Consortium. Ag1000G phase 1 AR2 data release. MalariaGEN. http://www.malariagen.net/data/ag1000g-phase1-AR2 (Date of Access: 19/12/2014) (2014).

[b64] DePristo M. A. . A framework for variation discovery and genotyping using next-generation DNA sequencing data Nat. Genet. 43, 491–498 (2011). 21478889 10.1038/ng.806 PMC3083463

[b65] The 1000 Genomes Project Consortium. An integrated map of genetic variation from 1,092 human genomes. Nature 491, 56–65 (2012). 23128226 10.1038/nature11632 PMC3498066

[b66] Danecek P. . The variant call format and VCFtools. Bioinformatics 27, 2156–2158 (2011). 21653522 10.1093/bioinformatics/btr330 PMC3137218

[b67] Turner D. S. qqman: An R package for visualizing GWAS results using QQ and Manhattan plots. BioRxiv, doi: 10.1101/005165 (2014).

[b68] Chang C. C. . Second-generation PLINK: rising to the challenge of larger and richer datasets. GigaScience 4, 7, doi: 10.1186/s13742-015-0047-8 (2015). 25722852 PMC4342193

[b69] Patterson N., Price A. L. & Reich D. Population structure and eigenanalysis. PLoS Genet. 2, e190, doi: 10.1371/journal.pgen.0020190 (2006). 17194218 PMC1713260

[b70] Martinez-Torres D. . Molecular characterization of pyrethroid knockdown resistance (*kdr*) in the major malaria vector *Anopheles gambiae* s.s. Insect Mol. Biol. 7, 179–184 (1998). 9535162 10.1046/j.1365-2583.1998.72062.x

[b71] Ranson H. . Identification of a point mutation in the voltage-gated sodium channel gene of Kenyan *Anopheles gambiae* associated with resistance to DDT and pyrethroids. Insect Mol. Biol. 9, 491–497 (2000). 11029667 10.1046/j.1365-2583.2000.00209.x

[b72] Janeira F. . A primer-introduced restriction analysis-polymerase chain reaction method to detect knockdown resistance mutations in *Anopheles gambiae*. J. Med. Entomol. 45, 237–241 (2008). 18402139 10.1603/0022-2585(2008)45[237:apracr]2.0.co;2

[b73] Weill M. . The unique mutation in ace-1 giving high insecticide resistance is easily detectable in mosquito vectors. Insect Mol. Biol. 13, 1–7 (2004). 14728661 10.1111/j.1365-2583.2004.00452.x

[b74] Simões M. J., Próspero M. I. & Ribeiro H. Optimização Da Técnica ELISA ‘Two Sites’ Utilizada Na Identificação de Refeições Sanguíneas de Mosquitos. Rev. Port. Doenc. Infec. 18, 225–229 (1995).

