# Supplementary material for: Massive introgression drives species radiation at the range limit of Anopheles gambiae
Source: Sci Rep. 2017 Apr 18;7:46451. doi: 10.1038/srep46451 (PMC5394460; doi:10.1038/srep46451)
Supplement: Supplementary Figures and Tables [file srep46451-s1.pdf]

Is massive introgression driving species radiation at the range limit of *Anopheles gambiae*?

José L. Vicente[1,*], Christopher S. Clarkson[2,*], Beniamino Caputo[3,*], Bruno Gomes[1], Marco Pombi[3], Carla A. Sousa[1], Tiago Antao[2,†], João Dinis[4], Giordano Bottà[3,5], Emiliano Mancini[3, ‡], Vincenzo Petrarca[3,6], Daniel Mead[7], Eleanor Drury[7], James Stalker[7], Alistair Miles[5,8], Dominic P. Kwiatkowski[5,7,8], Martin J. Donnelly[2], Amabélia Rodrigues[4], Alessandra della Torre[3], David Weetman[2,*] and João Pinto[1,*]

1. Global Health & Tropical Medicine, Instituto de Higiene e Medicina Tropical, Universidade Nova de Lisboa, Lisbon, Portugal;
2. Department of Vector Biology, Liverpool School of Tropical Medicine, Liverpool, United Kingdom;
3. Istituto Pasteur Italia-Fondazione Cenci-Bolognetti, Dipartimento di Sanità Pubblica e Malattie Infettive, Università di Roma "Sapienza", Rome, Italy;
4. Instituto Nacional de Saúde Pública, Ministério da Saúde Pública, Bissau, Guiné-Bissau;
5. Wellcome Trust Centre for Human Genetics, University of Oxford, Oxford, United Kingdom;
6. Istituto Pasteur Italia-Fondazione Cenci-Bolognetti, Dipartimento di Biologia e Biotecnologie "Charles Darwin", Università di Roma "Sapienza", Rome, Italy.
7. Wellcome Trust Sanger Institute, Hinxton, UK.
8. Medical Research Council Centre for Genomics and Global Health, University of Oxford, Oxford, UK.

* These authors contributed equally to this study.

Supplementary Figures and Tables

Supplementary Table 1| Microsatellites used in the study.

Chromosome	Locus	Repeat	Dye	Primers	T_A
X	X6U2[a]	$(CA)_{13}$	HEX	F*-TTGTTGCTCGGCTTGAAGTA<br>R-GAAGGAATCGAGGGTGCTCT	60
	X5D1[a]	$(CA)_{11}$	NED	F*-GGAAACCGACACCACAAAG<br>R-TGCCATTGAATGATGATGATG	55
	X678	$(TC)_{7}$	HEX	F*-CCTCTCCCCAGAATCGGTAC<br>R-AAGAGCAGAAACAACCGCAG	54
	X5C1[a]	$(GT)_{10}$	6-FAM	F*-TCGCTTCGACAAATCATCAC<br>R-GGGCGAAAATTCGTACAGAG	60
	X5B1[a]	$(CA)_{10}$	NED	F*-CAACAGCGAAAGGGTTATCG<br>R-CAGCAGAACATACACGCACA	62
	XH07	$(AT)_{3}(GT)_{7}$	HEX	F*-CACGATGGTTTTCGGTGTGG<br>R-ATTTGAGCTCTCCCGGGTG	54
	XH25	$(GT)_{9}$	6-FAM	F*-GCCGAAAACATTCCAACAGG<br>R-CAGTTATGTCGGCATGCTAC	54
	XH77	$(GT)_{10}$	6-FAM	F*-TGGGACTGTAAGTGTCTCCC<br>R-TATCAGTGAGGCCGAGTTGC	54
	X145	$(GT)_{11}$	HEX	F*-TGGTGGAATGTGAGACACAG<br>R-ATGATGGTCGATCCTTGTCC	54
3L	H577	(GT)16	6-FAM	F-TTCAGCTTCAGGTTGGTCTC<br>R*-GGGTTTTTTGGCTGCGACTG	56
	H758	(GT)11	6-FAM	F*-TGATTTGCCAGTTCTGCCAG<br>R-GTGATTGGAGTGGCTAGTGG	54
	H242	(GT)8	HEX	F*-TTCATTTCCACCGCAGCTGC<br>R-GGCGACACTCAATCCTTCC	56
	H45C[b]	(TG)4+7+4	HEX	F*-AAAAGTGGTGACCGAGTGAC<br>R- ATCTTCAACACTTCAGCACG	54
3R	H555	(GT)8	NED	F*-GCAGAGACACTTTCCGAAAC<br>R-TGTCAACCCACATTTTGCGC	54
	H119	(GT)6	HEX	F*-GGTTGATGCTGAAGAGTGGG<br>R-ATGCCAGCGGATACGATTCG	54
	H249	(GT)15	NED	F*-ATGTTCCGCACTTCCGACAC<br>R-GCGAGCTACAACAATGGAGC	54
	H059	(GT)9	HEX	F*-CCCCTATTAAACCCTGGACG<br>R-TGTTGTTGCCCTGCGTTACC	54
	H128	(GT)21	6-FAM	F*-CGGGACGGCTAGATAAAGCG<br>R-CCGGGCGACATAACCCACCC	56
	H093	(GT)4+7	NED	F*-TCCCCAGCTCACCCTTCAAG<br>R-GGTTGCATGTTTGGATAGCG	54

Dye: Applied Biosystems® fluorescent dye used to label the primer. * Primer fluorescently labeled; T_A: annealing temperature; [a] Stump *et al*. 2005. *Genetics* 169, 1509-1519, [b] Lehmann *et al.* 1998. *Mol. Biol. Evol.* 15, 264-276, remaining loci were described in Zheng *et al*. 1996. *Genetics* 143, 941-952.

Supplementary Table 2| Genetic diversity estimates per microsatellite locus and locality.

Locus		Quinhamel	Safim	Antula	Mansoa	Mandingará	Gambana	Comuda	Leibala	Mean
X6U2	$Ar_{(100)}$	8.1	9.2	8.3	8.6	9.7	6.2	11.2	14.4	9.4
	H_e	0.660	0.661	0.669	0.801	0.817	0.732	0.838	0.832	0.751
	F_{IS}	0.101	0.007	0.052	0.054	0.059	0.062	-0.009	-0.008	0.040
X5D1	$Ar_{(100)}$	7.0	5.7	5.0	7.6	7.5	6.2	6.3	6.5	6.5
	H_e	0.543	0.526	0.614	0.587	0.661	0.618	0.709	0.640	0.612
	F_{IS}	0.130	-0.084	0.107	0.122	-0.072	0.003	-0.070	0.008	0.018
X678	$Ar_{(100)}$	14.7	15.2	15.7	15.7	10.2	7.8	15.3	20.0	14.3
	H_e	0.805	**0.785**	0.796	0.834	0.646	0.531	0.870*	0.886	0.769
	F_{IS}	0.044	0.124	-0.054	0.170	0.240	0.068	**0.180**	0.041	0.102
X5C1	$Ar_{(100)}$	12.5	10.8	13.7	12.7	11.3	11.6	10.9	11.3	11.9
	H_e	0.825	0.801	0.819	0.843	0.842	0.835	0.819	0.846*	0.829
	F_{IS}	0.044	0.092	0.055	0.063	0.106	-0.077	**0.236**	0.175	0.087
X5B1	$Ar_{(100)}$	13.8	14.6	13.4	14.0	15.9	17.2	15.0	12.0	14.5
	H_e	0.851	0.810	0.817	**0.836***	0.865	0.869	0.846	0.799	0.837
	F_{IS}	0.031	0.138	0.019	**0.346**	0.102	0.023	-0.012	0.112	0.095
XH07	$Ar_{(100)}$	8.1	8.2	7.8	7.9	10.2	7.9	8.2	7.4	8.2
	H_e	0.741	0.686	0.653	0.701	0.711	0.684	0.727*	**0.735***	0.705
	F_{IS}	-0.009	0.233	0.095	0.172	0.157	0.151	**0.261**	**0.410**	0.184
XH25	$Ar_{(100)}$	8.2	8.4	8.1	7.8	7.3	7.0	8.4	8.5	8.0
	H_e	0.720	0.730	0.714	0.764*	0.707	0.734	0.770	0.743	0.735
	F_{IS}	-0.038	0.102	- 0.008	0.199	0.130	0.023	0.102	0.104	0.077
XH77	$Ar_{(100)}$	8.6	8.5	8.8	11.5	9.5	8.4	9.6	8.9	9.2
	H_e	0.779	0.803	0.797	0.822	0.805	0.811	0.790	0.797	0.801
	F_{IS}	0.041	0.036	-0.025	-0.040	-0.009	-0.019	-0.057	0.110	0.005
X145	$Ar_{(100)}$	6.9	7.8	7.1	9.3	7.7	7.2	7.8	7.4	7.7
	H_e	0.743	0.743	0.727	0.745	0.725	0.740	0.739	0.717	0.735
	F_{IS}	-0.066	0.147	-0.020	-0.061	-0.057	0.031	-0.056	0.011	-0.009
Mean	$Ar_{(100)}$	9.8	9.8	9.8	10.6	9.9	8.8	10.3	10.7	
X-loci	H_e	0.741	0.727	0.734	0.770	0.753	0.728	0.790	0.777	
	F_{IS}	0.031	0.088	0.025	0.114	0.073	0.029	0.064	0.107	

$Ar_{(100)}$: Allele richness. H_e: expected heterozygosity (in bold: significant heterozygote deficit). F_{IS}: inbreeding coefficient (in bold: significant value). Asterisks indicate presence of null alleles determined by Micro-Checker. X-loci: chromosome-X microsatellites; 3-loci: chromosome-3 microsatellites.

Supplementary Table 2 (continued).

Locus		Quinhamel	Safim	Antula	Mansoa	Mandingará	Gambana	Comuda	Leibala	Mean
H093	$Ar_{(100)}$	15.2	16.5	14.0	13.8	14.5	14.2	13.2	11.6	14.1
	H_e	0.843*	**0.836***	**0.822**	0.779	0.842	0.825*	0.841*	0.816	0.825
	F_{IS}	**0.197**	**0.242**	0.163	0.215	0.142	**0.205**	0.177	0.091	0.179
H128	$Ar_{(100)}$	22.0	23.3	25.7	19.9	21.5	21.7	22.9	22.3	22.4
	H_e	0.923	0.930	0.937*	0.923*	0.929*	**0.915***	0.918	0.925	0.925
	F_{IS}	-0.036	0.018	0.094	**0.180**	**0.194**	**0.142**	0.069	0.082	0.093
H059	$Ar_{(100)}$	8.9	9.8	9.3	8.6	7.7	9.2	10.4	8.3	9.0
	H_e	0.797	0.808	0.797	0.818	0.779	0.759	0.806	0.814	0.797
	F_{IS}	0.049	0.042	0.082	0.153	0.191	0.136	0.100	0.115	0.109
H249	$Ar_{(100)}$	15.5	16.0	18.0	15.1	12.9	19.7	16.0	14.2	15.9
	H_e	0.871	0.883	0.861	0.870	0.874	0.865	0.854	0.873	0.869
	F_{IS}	0.093	-0.023	0.038	0.092	0.139	**0.136**	0.061	0.040	0.072
H119	$Ar_{(100)}$	14.6	15.4	14.6	15.0	16.1	14.5	14.6	13.7	14.8
	H_e	0.875	0.858	0.850	0.852	0.879	0.886*	0.865	0.833	0.862
	F_{IS}	0.033	0.023	0.026	-0.003	0.144	0.146	0.099	0.058	0.066
H555	$Ar_{(100)}$	9.0	12.6	9.6	9.8	11.3	9.1	9.9	11.6	10.4
	H_e	0.827	0.815	0.810	0.823*	0.832	0.837	0.815	0.821	0.822
	F_{IS}	0.057	-0.082	0.078	0.198	-0.017	-0.026	0.056	-0.022	0.030
H577	$Ar_{(100)}$	11.4	11.6	13.8	9.3	11.8	11.0	12.6	10.8	11.5
	H_e	0.686	0.709	**0.761**	0.574	0.651	0.642	0.684	0.636	0.668
	F_{IS}	-0.074	-0.016	0.144	-0.069	- 0.016	0.041	-0.028	-0.014	-0.004
H758	$Ar_{(100)}$	14.8	18.4	15.4	18.0	14.6	15.6	14.8	16.7	16.0
	H_e	**0.896***	0.911	0.901	0.907*	0.894	**0.892***	0.894*	0.881*	0.897
	F_{IS}	**0.179**	**0.141**	0.085	**0.183**	0.158	**0.143**	**0.168**	**0.137**	0.149
H242	$Ar_{(100)}$	7.4	8.8	8.2	9.2	7.3	7.1	7.9	9.0	8.1
	H_e	0.648	**0.739**	0.620	0.668	0.744	0.651	0.686	0.671	0.678
	F_{IS}	0.102	0.142	0.098	0.156	0.133	0.194	0.071	-0.009	0.111
H45C	$Ar_{(100)}$	6.9	7.2	6.3	7.6	7.0	7.2	7.6	8.2	7.3
	H_e	0.795	0.782	0.769	0.746	0.788	0.746	0.770	0.775	0.771
	F_{IS}	-0.024	0.125	0.057	0.180	0.044	0.066	0.001	0.043	0.062
Mean	$Ar_{(100)}$	12.6	14.0	13.5	12.6	12.5	13.0	13.0	12.6	
3-loci	H_e	0.816	0.827	0.813	0.796	0.821	0.802	0.813	0.804	
	F_{IS}	0.058	0.061	0.087	0.129	0.111	0.118	0.077	0.052	
Mean All loci	$Ar_{(100)}$	11.2	12.0	11.7	11.6	11.3	11.0	11.7	11.7	
	H_e	0.780	0.780	0.776	0.784	0.789	0.767	0.802	0.792	
	F_{IS}	0.045	0.074	0.057	0.122	0.093	0.076	0.071	0.078	

Supplementary Table 3| Accession numbers, Mean sequencing depth (autosomes), karyotype and *kdr* genotypes for the individuals sequenced.

Individual code	ERS Accession	Mean sequencing depth	Country	Region	Species (IGS/SINE assays)	2L Karyotype	2Rb Karyotype	2Rd Karyotype	*kdr* genotype
AJ0001-C	ERS040121	29,8	Guinea Bissau	Leibala	*A. gambiae*	+/+	+/+	+/+	L/L
AJ0007-C	ERS040133	26,6	Guinea Bissau	Leibala	*A. gambiae*	+/+	+/+	+/+	L/L
AJ0009-C	ERS040129	31,5	Guinea Bissau	Leibala	*A. gambiae*	a/a	b/+	+/+	L/L
AJ0011-C	ERS040122	29,8	Guinea Bissau	Leibala	*A. gambiae*	a/a	b/+	+/+	L/L
AJ0013-C	ERS040137	32,7	Guinea Bissau	Safim	*A. gambiae*	+/+	+/+	d/d	L/L
AJ0014-C	ERS040120	20,8	Guinea Bissau	Safim	*A. gambiae*	a/a	b/b	+/+	L/L
AJ0016-C	ERS040130	33,4	Guinea Bissau	Safim	*A. gambiae*	+/+	+/+	d/+	L/L
AJ0018-C	ERS040123	22,7	Guinea Bissau	Safim	*A. gambiae*	+/+	+/+	d/d	L/L
AJ0020-C	ERS040132	25,4	Guinea Bissau	Safim	*A. gambiae*	+/+	+/+	+/+	L/L
AA0006-C	ERS012670	24,7	Ghana	Greater Accra	*A. gambiae*	-	-	-	F/F
AA0007-C	ERS012671	21,9	Ghana	Greater Accra	*A. gambiae*	-	-	-	F/F
AA0008-C	ERS012672	24,9	Ghana	Greater Accra	*A. gambiae*	-	-	-	F/F
AA0009-C	ERS012673	17,8	Ghana	Greater Accra	*A. gambiae*	-	-	-	F/F
AJ0043-C	ERS242776; ERS254362	27,9	Guinea Bissau	Antula	*A. gambiae*	-	-		L/L
AJ0047-C	ERS242791; ERS254358	20,2	Guinea Bissau	Antula	*A. gambiae*	-	-	-	L/L
AJ0059-C	ERS242792; ERS254321	31,9	Guinea Bissau	Antula	*A. gambiae*	-	-	-	L/L
AJ0061-C	ERS242801; ERS254310	25,7	Guinea Bissau	Antula	*A. gambiae*	-	-	-	L/L
AJ0071-C	ERS224332	34,1	Guinea Bissau	Antula	*A. gambiae*	-	-	-	L/L
AJ0076-C	ERS224284	35,4	Guinea Bissau	Antula	*A. gambiae*	-	-	-	L/L
AJ0085-C	ERS242803; ERS254298	87,2	Guinea Bissau	Antula	*A. gambiae*	-	-	-	L/L
AJ0096-C	ERS224313	35,1	Guinea Bissau	Antula	*A. gambiae*	-	-	-	L/L
AJ0098-C	ERS224312	33,6	Guinea Bissau	Antula	*A. gambiae*	-	-	-	L/L
AJ0100-C	ERS224272	32,5	Guinea Bissau	Antula	*A. gambiae*	-	-	-	L/L
AJ0107-C	ERS224805	29,7	Guinea Bissau	Antula	*A. gambiae*	-	-	-	L/L
AJ0113-C	ERS224286	31,7	Guinea Bissau	Antula	*A. gambiae*	-	-	-	L/L

Supplementary Table 4| Blood meal identification by ELISA according to STRUCTURE genetic clusters.

IgG-positive	*A. coluzzii* (13)	*A. gambiae*-inland (34)	*A. gambiae*-coast (65)	Admixed (23)
Human	38.5	82.4	24.6	34.8
Bovine	30.8	5.9	43.1	52.2
Porcine	15.4	8.8	9.2	0.0
Canine	7.7	0.0	4.6	4.3
Bovine/porcine	0.0	0.0	4.6	0.0
Human/porcine	0.0	0.0	4.6	0.0
Human/bovine	0.0	0.0	0.0	4.3
Human/caprine	0.0	0.0	1.5	0.0
Caprine/porcine	0.0	0.0	0.0	4.3
Other/negative	7.7	2.9	7.7	0.0

In parenthesis: number of blood meals analyzed for each genetic cluster. Eight individuals with blood meal from Antula (6 *A. gambiae*; two admixed by IGS/SINE) were not included in microsatellite analysis.

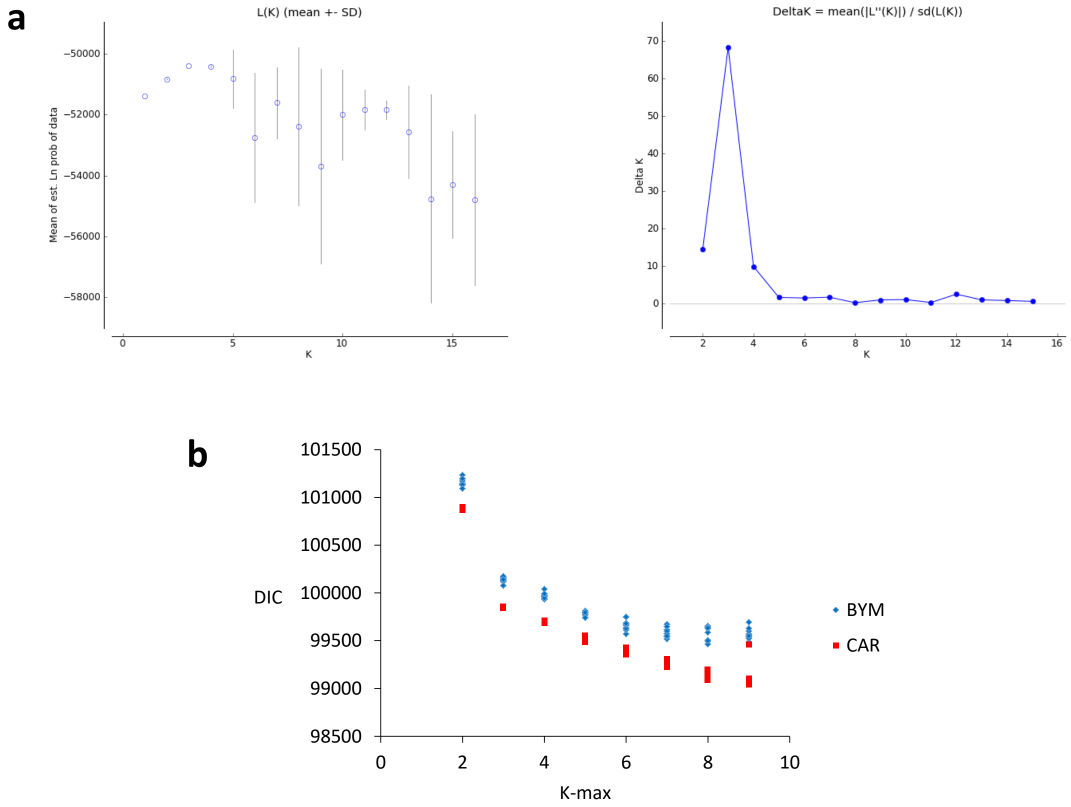

Supplementary Figure 1| Inference of the number of clusters obtained from microsatellite-based Bayesian clustering (STRUCTURE) and spatially explicit (TESS) analyses (**a**) Plots for inferring *K* from STRUCTURE. Left plot is for the ln[Pr(X|K)] (Pritchard *et al.* 2000. *Genetics* 155, 945-959) and right plot for the ΔK (Evanno *et al.* 2005. *Mol. Ecol.* 14, 2611-2620). (**b**) Deviance Information Criterion (DIC) plot for BYM and CAR models to infer the optimal number of clusters obtained by TESS (Chen *et al.* 2007. *Mol. Ecol. Notes* 7, 747-756).

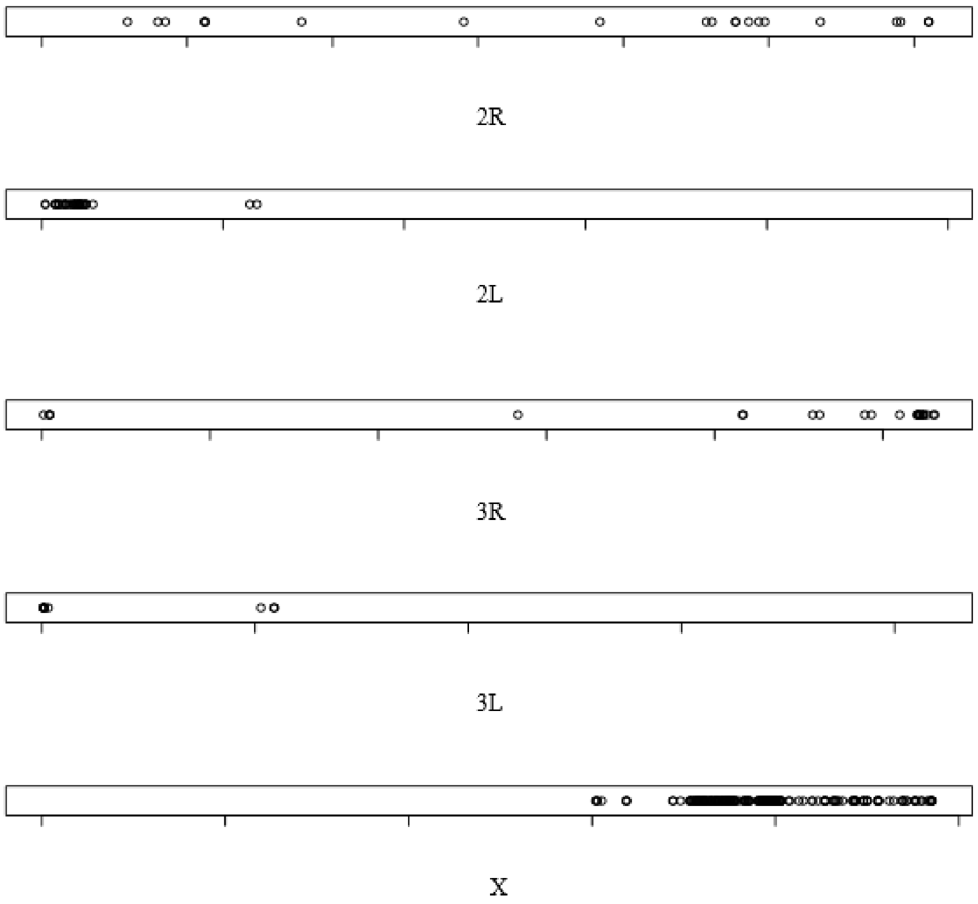

Supplementary Figure 2| Distribution of ancestry informative markers (AIMs). Each bar represents a chromosome within which the relative location of the SNPs used in the AIM analysis is marked by circles.

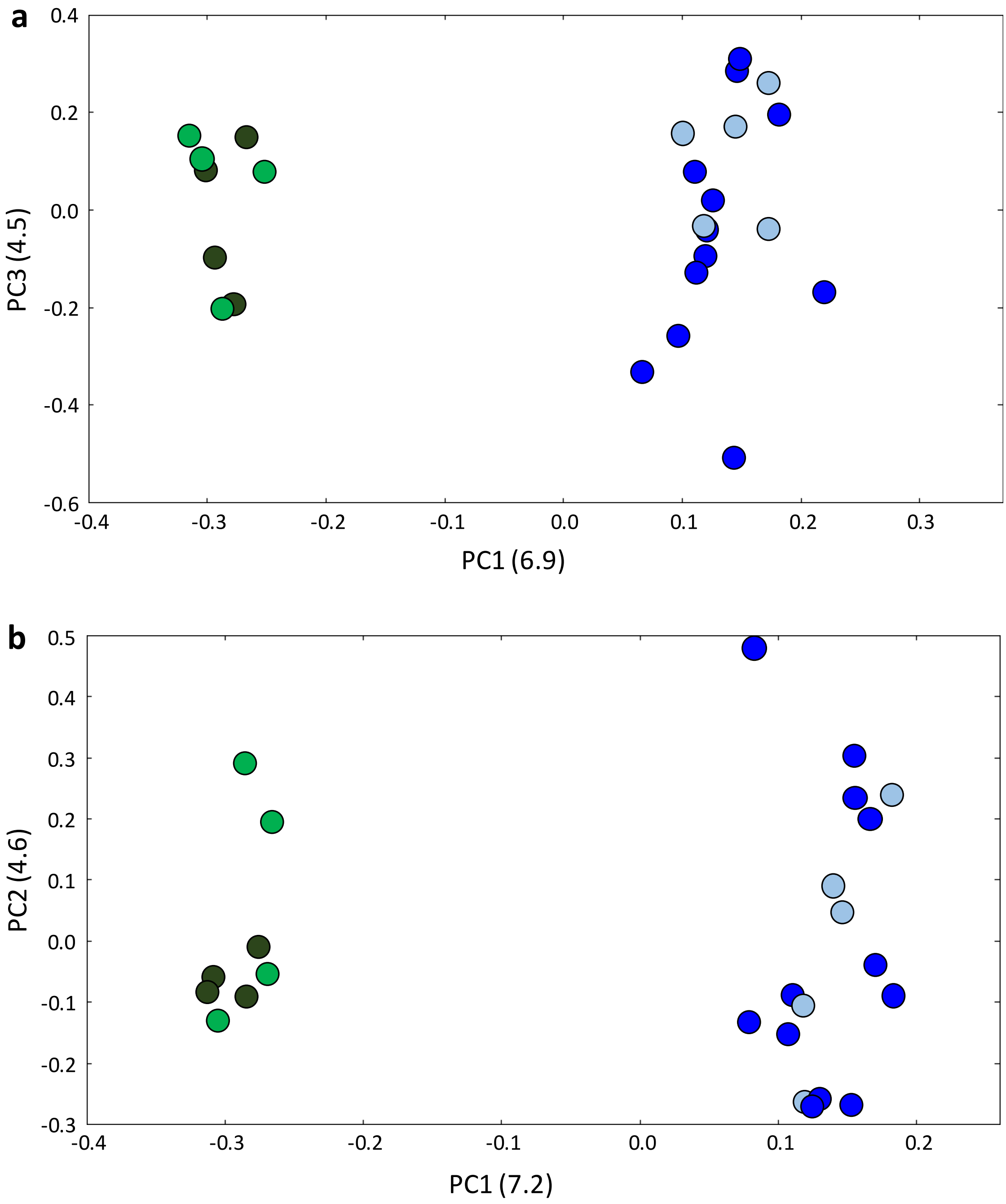

Supplementary Figure 3| Principal Components Analysis for whole genome sequenced *A. gambiae*. (**a**) Plot between principal components 1 and 3 for chromosome 3L variants. (**b**) Plot between principal components 1 and 2 for chromosome 3R variants. Sequenced sample are labelled as follows: Antula (blue), Safim (light blue), Leibala (green) and Accra-Ghana (dark green).

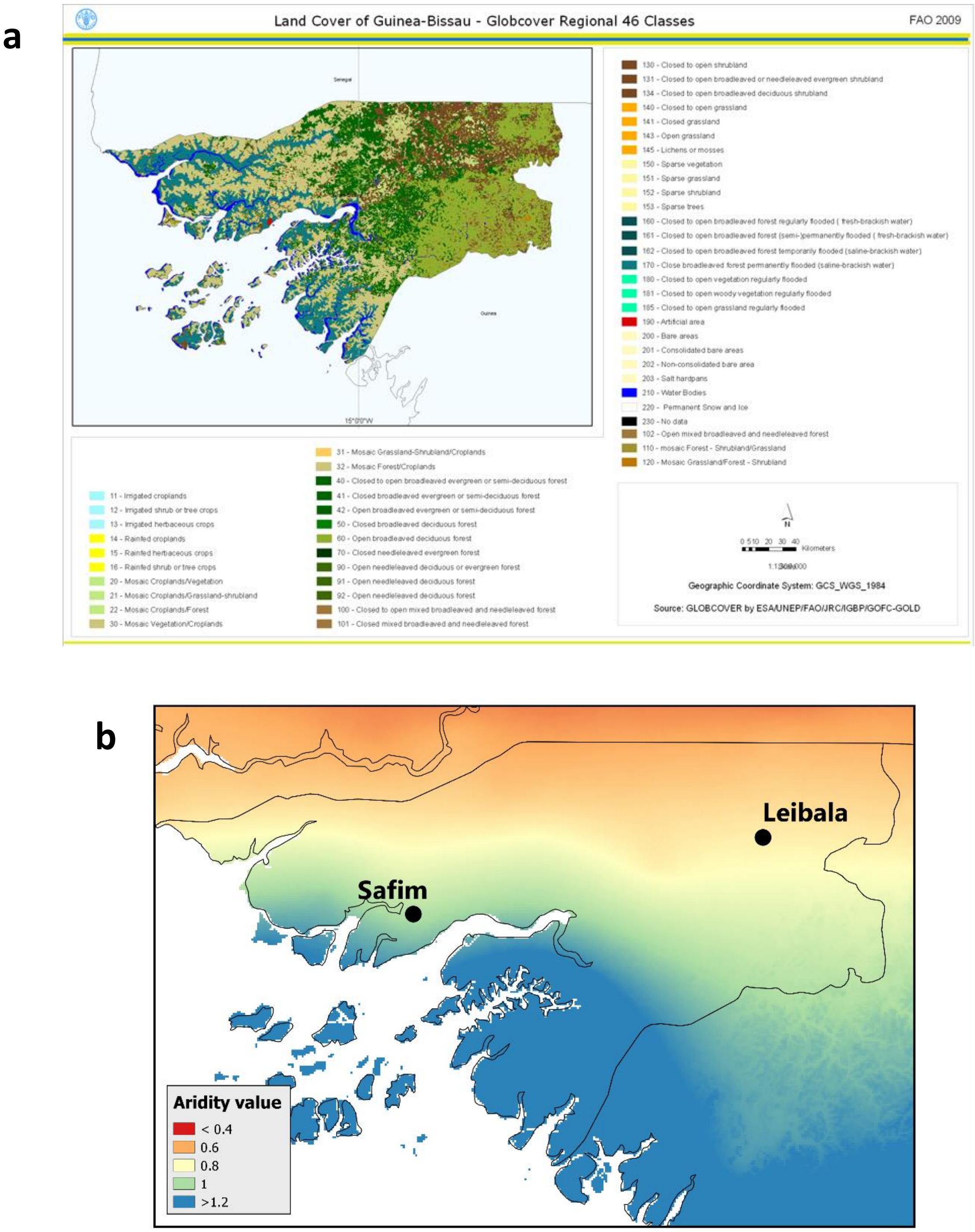

Supplementary Figure 4| Landcover and aridity maps of Guinea Bissau. (**a**) GlobCover 2009 land cover map of Guinea Bissau. Copyright notice: © ESA 2010 and UCLouvain. Available at: http://www.fao.org/geonetwork/srv/en/metadata.show?id=37189&currTab=simple. (**b**) aridity map for Guinea Bissau based on the CGIAR-CSI Global Aridity Index database. The map shows location of the two sites for which chromosomal data are available. Values are the mean Aridity Index from the 1950-2000 period at 30' spatial resolution (Trabucco, A., & Zomer, R.J. 2009. *Global Potential Evapo-Transpiration (Global-PET) and Global Aridity Index (Global-Aridity) Geo-Database*. CGIAR Consortium for Spatial Information. Available from the CGIAR-CSI GeoPortal at: http://www.csi.cgiar.org)
